# The Role of Intensifying Precipitation on Coastal River Flooding and Compound River‐Storm Surge Events, Northeast Gulf of Mexico

**DOI:** 10.1029/2020WR029363

**Published:** 2021-11-10

**Authors:** S. L. Dykstra, B. Dzwonkowski

**Affiliations:** ^1^ Department of Marine Sciences University of South Alabama Dauphin Island Sea Lab Dauphin Island AL USA; ^2^ Dauphin Island Sea Lab Dauphin Island AL USA; ^3^ Now at School of the Earth, Ocean and Environment University of South Carolina Columbia SC USA

**Keywords:** coastal flooding, compound event, climate change, coastal hydrology, ENSO, Gulf of Mexico

## Abstract

Destructive coastal floods are commonly increasing in frequency and may be caused by global precipitation intensification. Such connections through climate, watershed, and river processes are poorly understood because of complex interactions in transitional fluvial‐marine environments where flooding is caused by rivers, marine storm surge, or both in compound events. To better understand river floods along the fluvial‐marine transition, we study watersheds of the northeastern Gulf of Mexico using long‐term observations. Results show intensifying precipitation decreased precipitation‐discharge lag times, increasing river‐flood frequency and the likelihood of compound events in fluvial‐marine transitions. This reduction in lag time occurred when the Atlantic Multidecadal Oscillation and El Niño Southern Oscillation began strongly affecting river discharge through the advection of moist air, intensifying precipitation. Along the fluvial‐marine transition, compound events were largest in inland reaches. However, for inland reaches, compound event water levels did not exceed the floods caused solely by river flooding, the largest flood hazard in these systems. Our results demonstrate precipitation and river discharge play critical roles in coastal flooding and will likely escalate flooding as the climate continues to warm and intensify precipitation.

## Key Points

- Long‐term observations show a climate induced precipitation intensification shortened the time between precipitation and coastal discharge
- Teleconnections produced positive and negative ENSO‐discharge correlations, which oscillated slowly at a 60‐year period
- Results from the Gulf of Mexico watersheds suggest global warming is increasing the frequency of compound river‐marine flooding

## 1 Introduction

**Figure 1:**
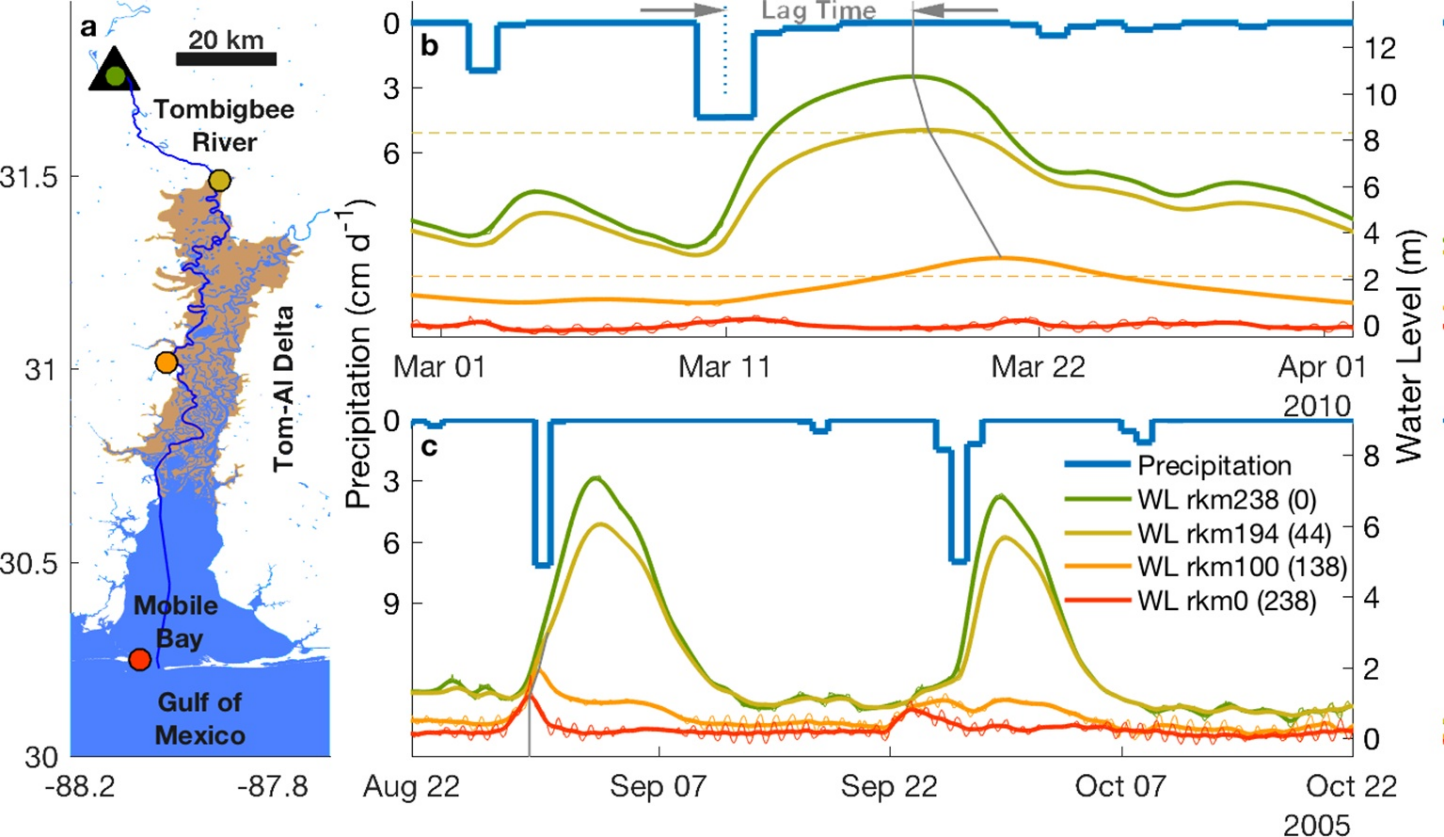
Map of the study region with associated water level events. (a) A map of the Tombigbee River‐Mobile Bay fluvial‐marine transition (dark blue line) spanning 238 km from the Coffeeville Lock and Dam (triangle) through the Tombigbee‐Alabama Delta (brown) to the Gulf of Mexico. (b) Example of an extensive river flooding event that propagates seaward (i.e., a river flood wave) across the fluvial‐marine transition. (c) Examples of two river wave events compounding with landward propagating storm surge waves in the fluvial‐marine transition. Subtidal water level (WL) time series in b and c are color coded to the station location in panel (a) and are labeled by river kilometers (rkm) from the mouth (dam). Time series of Tombigbee‐Alabama Watershed precipitation (blue line) are also shown in panel (b and c) from which the lag‐time between an extreme precipitation event (vertical dashed line) and a peak water level (gray line) can be determined (i.e., precipitation‐discharge time lag). The gray line between peak water levels shows wave propagation from which the slope captures attenuation. In panel (c), the initiation and landward propagating storm surge wave is similarly shown with a gray line at the bottom of the plot. In panel (b), flood stage conditions occurred when water level exceeded levee/bank elevations marked for station locations at rkm 100 and 194 (horizontal dashed lines). The thin colored lines, where visible in panel (b and c), are hourly water level observations capturing the tidal signal.

Coastal floods are common expensive natural disasters, associated with dense coastal populations and wide‐reaching disruptions to global transportation (e.g., ports; Hendry et al., 2019; Moftakhari et al., 2017). Infrastructure and investments are concentrated where rivers reach the sea and floods can come from both sources making risk management difficult (AghaKouchak et al., 2020; Devlin et al., 2017). This transition from fluvial to marine environments can extend for hundreds of kilometers, from the tidal limit to regions of freshwater influence on the shelf, and contain distinctive changes in geometry, circulation, and water quality (e.g., Figure 1a; Dalrymple & Choi, 2007; Gugliotta et al., 2017). These transitional environments support natural ecosystems that depend on coastal river flooding and are critical to global biogeochemical cycles, shoreline protection, and coastal productivity (Bauer et al., 2013; Hopkinson & Vallino, 1995). Flooding along the fluvial‐marine transition is primarily characterized by marine processes, such as storm surge from strong winds (e.g., Spicer et al., 2019). This may be due to the poor understanding of river flooding beyond the traditional field of fluvial hydrology, which typically ends where tidal influence begins. River flooding in the fluvial‐marine transition (herein coastal river flooding, a form of coastal flooding) poses critical risks to coastal communities, leaving them vulnerable and unprepared.

River flooding is primarily controlled by precipitation and watershed hydrology, factors that can change from event to event or over long periods of time (Gericke & Smithers, 2014; Shiklomanov et al., 2007; Turner‐Gillespie et al., 2003; Villarini et al., 2009; Williams, 1978; Woltemade & Potter, 1994). Intense precipitation events saturate soils, increasing runoff. Events with larger runoff coefficients (i.e., discharge volume/precipitation volume) have shorter precipitation‐discharge lag times (i.e., time between precipitation and discharge events; e.g., Figure 1b), larger peak discharges, and higher water levels (Gericke & Smithers, 2014). These precipitation effects on discharge dissipate through time, reducing in a downstream direction and as watershed size increases (e.g., Fleischmann et al., 2016; Turner‐Gillespie et al., 2003; Woltemade & Potter, 1994). Thus, precipitation events propagate faster to a downstream location as rainfall intensifies and the size of watersheds become smaller (Gericke & Smithers, 2014; McCuen & Spiess, 1995, and references therein).

Over long periods of time, precipitation volume and intensity can change with climate oscillations, periodically modifying the risk of extreme flooding. This has been observed in the Amazon Basin due to a modulation of the Walker circulation by the Atlantic Multidecadal Oscillation (AMO) and El Niño Southern Oscillation (ENSO; Barichivich et al., 2018). However, in some Amazonian subwatersheds, episodic floods associated with ENSO have not occurred since the 1960s, indicating a change in climate (Aalto et al., 2003). Separating the impacts of climate modes from climate change is extremely challenging in observational data due to limited durations of time series (Axelson et al., 2009). Identifying non‐stationary processes associated with climate patterns are critical for predicting extreme floods as traditional flood risk methods rely on stationary assumptions (Barichivich et al., 2018; Enfield et al., 2001; Grinsted et al., 2004).

The most widely observed non‐stationary change in climate is global warming, a process increasing the moisture content of the air by ∼7%–8% per degree celsius, resulting in a global precipitation intensification of similar magnitude (Allan & Soden, 2008; Easterling et al., 2017; Kirchmeier‐Young & Zang, 2020). As precipitation events become larger, runoff increases exponentially (e.g., Lemma et al., 2018). The intensification of precipitation in the central United States is increasing the frequency of river flooding in small watersheds (<5,000 km^2^) with surprisingly little change in the magnitude of the largest annual events (Kunkel et al., 1999; Mallakpour & Villarini, 2015). If and how precipitation intensification affects flooding in large watersheds (e.g., above the global median: 23,000 km^2^; Frasson et al., 2019) or coastal river flooding remains unknown.

The marine influence makes river flooding along fluvial‐marine transitions distinct from traditionally studied inland river flooding. Often, the same low‐pressure system that produces intense precipitation and elevated river water levels also produces a storm surge event (Bevacqua et al., 2019; Ganguli & Merz, 2019; Hendry et al., 2019; Talke et al., 2021). In the fluvial‐marine transition, these river and surge events can temporally and spatially coincide in a compound event, increasing the overall event magnitude and likelihood of flooding. The dynamics of these compound fluvial‐marine events (herein compound events) are associated with the overlapping interactions of seaward propagating river flood waves and landward propagating surge waves (e.g., Figure 1c; AghaKouchak et al., 2020; Ganguli & Merz, 2019; Spicer et al., 2019). River flood wave celerity (i.e., wave speed) generally scales with river event size, which is associated with precipitation event size (Turner‐Gillespie et al., 2003; Wolff & Burges, 1994). Storm surge waves, generated by coastal setup, are fast, suggesting compound flooding is nearly simultaneous along the fluvial‐marine transition, allowing a common practice of capturing compound floods in one location per system (e.g., Bevacqua et al., 2019; Hendry et al., 2019; Moftakhari et al., 2017; Wahl et al., 2015; Ward et al., 2018). However, recent work shows river waves slow down along fluvial‐marine transitions (Dykstra & Dzwonkowski, 2020a), suggesting compound events have temporal and spatial variability. Observations of compound events show river events usually follow storm surge events and are distinct without compounding as watersheds become larger, that is, exceed 5,000 km^2^ (Bevacqua et al., 2020; Hendry et al., 2019). This suggests compounding is niche to very small watersheds and have become the focus of compound flooding research (e.g., Bevacqua et al., 2019; Wahl et al., 2015). However, if precipitation‐discharge lag time decreases with the global precipitation intensification and causes river flood waves to propagate faster, the risk of compound flooding could increase in all fluvial‐marine transitions.

**Figure 2:**
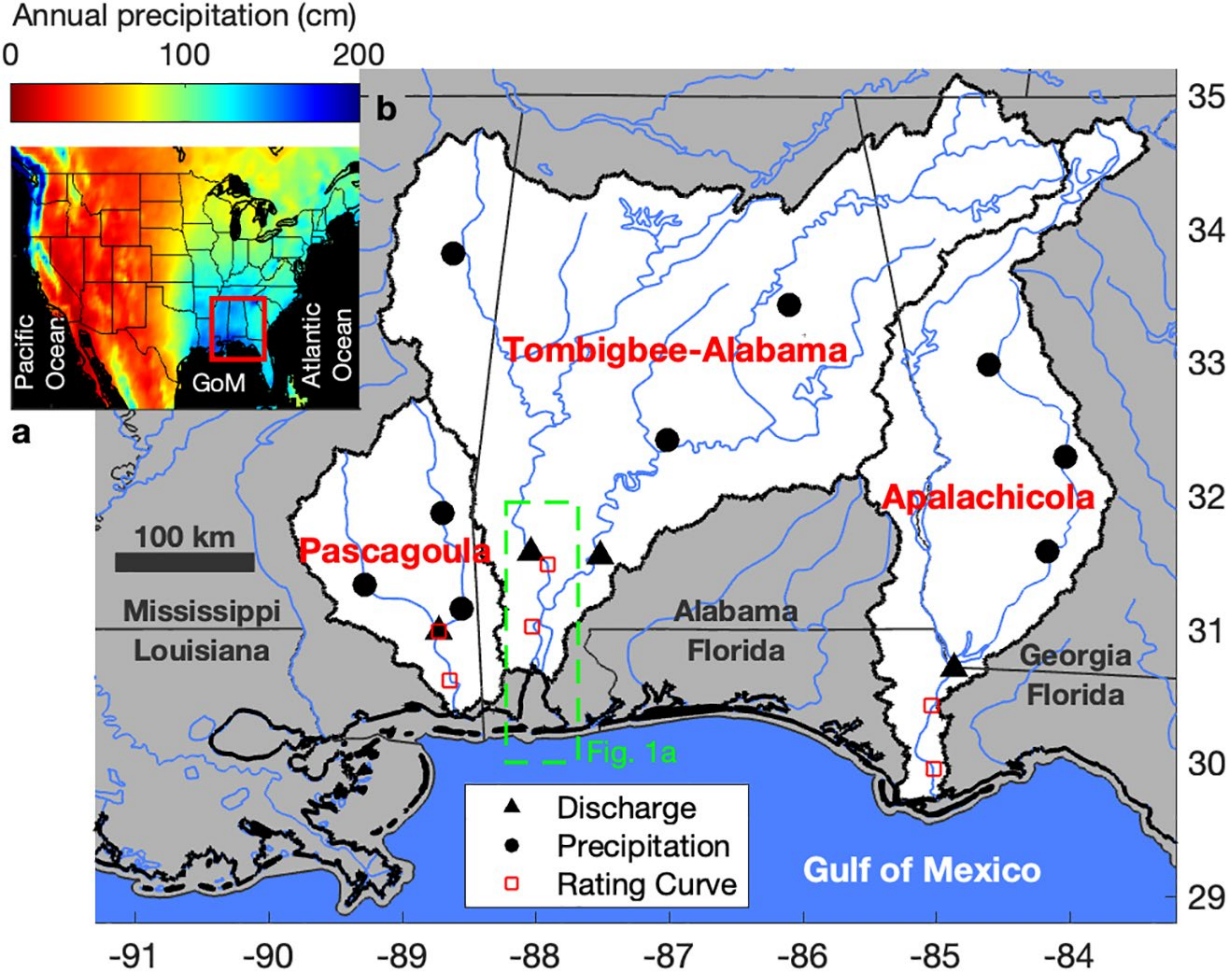
Northeast Gulf of Mexico (GoM) watersheds map with associated precipitation. (a) The annual precipitation of the continental United States (1891–2016) includes an outline of subfigure (b) (red square). (b) Studied watersheds are white and have rivers (blue lines) that flow into the northeast GoM.

Here, we investigate if precipitation intensification has increased the frequency and magnitude of coastal river flooding and what implications this has for compound events. We use long‐term observations to identify (a) changes through time in coastal river flooding, (b) sources causing the changes, and lastly, (c) the associated implications by observing individual events. To conduct the study, we chose fluvial‐marine transitions with watersheds that demonstrated an intensification of precipitation without a significant change in the annual precipitation volume. The systems were also chosen to demonstrate the affects of precipitation intensifications are applicable to large watersheds and are representative of the global median size and larger watersheds (i.e., >23,000 km^2^; Frasson et al., 2019): the Pascagoula, Tombigbee‐Alabama (Tom‐Al) and Apalachicola watersheds (23,860, 115,440, and 50,110 km^2^, respectively; Figure 2b). Additionally, the chosen systems have a high spatial consistency of mean precipitation and include the largest undammed waterway in the continental United States, the Pascagoula River (Figure 2a; Nilsson et al., 2005).

## 2 Materials and Methods

### 2.1 Data Sources and Data Sets

To observe changes in precipitation and coastal discharge, long‐term observational data (1905–2019 and 1930–2019, respectively; all years refer to hydro‐years, i.e., October–September) were accessed from NOAA and USGS. NOAA data included daily observed and gridded mean precipitation (Global Historical Climatology Network and Global Precipitation Climatology Center, respectively), as well as climate indices (Global Climate Observing System) and water levels (Tides & Currents). USGS data included daily discharge and hourly water levels (National Water Information System) as well as earth surface elevations, watershed delineations (Watershed Boundary Dataset), modeled historical land cover (Wieczorek et al., 2019), and rating curves (Rating Curve Builder). Curves for the Tom‐Al came from Dykstra and Dzwonkowski (2020a). A list of all stations, climate indices, and URLs to access the data, can be found in Table S1 in Supporting Information S1, as well as a flowchart of the methods (Figure S1 in Supporting Information S1).

Long‐term daily records were used to calculate one precipitation and one discharge time series for each of the three watersheds. In each watershed, three precipitation records (>94% complete) were averaged and multiplied by the watershed area for a complete precipitation volume record. For discharge, terminal long‐term measurements (>99% complete) were extrapolated by area for a total watershed discharge following Dykstra and Dzwonkowski (2020a); $Q_{w}=Q_{m}/k$, where *Q*_*m*_ is measured discharge, *k* is the fraction of watershed area represented by the measurement(s), that is measured area divided by total area.

At the terminus of each watershed, along the fluvial‐marine transition, flooding conditions and the frequency of floods were identified. The dynamics of river, surge, and tidal waves were observed using water levels, capturing celerity with the time between wave peaks and amplitude with the change in peak elevation (Dykstra & Dzwonkowski, 2020a; Turner‐Gillespie et al., 2003). To demonstrate these dynamics, we chose a representative fluvial‐marine transition, the Tom‐Al, and time periods when extreme precipitation events cooresponded with good water level coverage.

### 2.2 Statistical Analysis

Long‐term measurements were analyzed with consistent methods across the Pascagoula, Tom‐Al, and Apalachicola watersheds to capture changes in coastal flooding. Because the fluvial‐marine transition geometry is affected by marine forces, flooding was determined locally as any discharge event exceeding bankfull elevation for a given stream reach, following Williams (1978; method 4). Bankfull elevation was identified by averaging local levee elevations for each side of a river and taking the lowest. To reduce potential bias of a given reach (e.g., geomorphology changes), two reaches in each stream were used to determine bankfull conditions, at stations landward and seaward of the most commonly observed tidal limit. Each bankfull elevation was converted to bankfull discharge using a rating curve so flooding frequency through time only changed with discharge and not sea level.

Discharge frequency was analyzed using two different approaches. The first frequency method, continuous wavelet transformations, captures changes over time in a time‐frequency domain. The analysis followed Grinsted et al. (2004) with a Mortlet‐type wave and a non‐dimensional frequency scale of 6. This method calculates energy as a wavelet power spectrum normalized by variance, significance at a 95% confidence interval, and is commonly used for discharge (e.g., Axelson et al., 2009; Tongal et al., 2017; for more details see sources). To identify when event frequency changed, at each period, the number of significant events each year was tested using a non‐parametric Pettitt Test (e.g., Villarini et al., 2009; Tongal et al., 2017). Because discharge has red noise, to more clearly visualize the relative changes through time at each period, the wavelet power spectrum was normalized by period (in addition to variance). The second frequency approach identified flooding event probability and recurrence intervals using a Generalized Pareto distribution (GPD) following Castillo and Hadi (1997). The GPDs were fit to a partial duration series of discharge events using maximum likelihood estimate by employing the Matlab™ command gpfit. Each series was populated using exceedances over a threshold with events separated by at least a day (i.e., declustering period), an approach used for discharge in similar coastal plain environments (e.g., Sweet & Geratz, 2003). A GPD threshold is often determined by systematically increasing the threshold until the parameters (shape and scale) converge (e.g., Talke et al., 2018), but this method produced thresholds lower than mean discharge in all analyses and biased the GPDs to small events. To better represent flooding events, the threshold was set to the lowest bankfull discharge observed in each watershed, which also enabled flooding recurrence to be calculated at two bankfull discharges per system. GPD confidence intervals (95%) were calculated using a bootstrap resampling method of the data to generate 1,000 curves with Matlab™ commands gpfit and bootstrp (Castillo & Hadi, 1997; Das et al., 2016; Ralston et al., 2018). Additionally, the GPD models were compared to a rank order probability of the partial duration series (i.e., event order/number of events; e.g., Langbein, 1949). In each watershed, each frequency analysis was contrasted for the first and last 30 years (1930–1959 and 1990–2019, respectively), closely corresponding to the warm AMO periods (1928–1962 and 1996–2019, respectively).

Identifying the cause of increased coastal river flooding was done by analyzing interannual changes in watershed hydrology using the Pettitt Test. For precipitation and discharge volume, annual measurements were summed to evaluate interannual variability and calculate runoff coefficients. For timing, precipitation‐discharge lag times were calculated by cross correlating annual hydro‐year hyetographs and hydrographs, the recommend method of Gericke and Smithers (2014). In each hydrograph, to only capture events and reduce the annual influence of baseflow, hydrographs were first high‐pass filtered using a Lanczos filter where negative values were replaced with 0. Because results were nearly insensative for window sizes ∼1.5–4 months, 2‐month windows were used. The offset with the highest correlation was the annual precipitation‐discharge lag time. For precipitation intensity, an event was defined as any mean watershed precipitation exceeding the measurement threshold of 0.5 mm of rain per day and separated by a day below the threshold. Because precipitation was skewed in all watersheds with half the volume in the upper 81st–83rd percentile of events, the annual 82nd percentile was used to capture the overall precipitation intensity. Lastly, for land cover, modeled outputs from Total Upstream Coverage (TOT) were used to capture decadal averages for each watershed from the 1940s to 1990s, covering the period of significant changes in lag time and discharge frequency.

To explain long‐term changes in watershed hydrology, precipitation and discharge were tested using Kendall's rank correlation (*τ*) with 14 climate indices. Because only the AMO and ENSO had significant and consistant correlations with both precipitation and discharge (*p* < 0.1, *R*‐values positive or negative; Table S2 in Supporting Information S1), the relationships were tested for changes through time with moving‐window linear correlations (Enfield et al., 2001). Following Enfield et al. (2001), data were low‐pass filtered, correlated with a window twice the filter window length, and the confidence interval of the serially correlated results were determined using the method of Ebisuzaki (1997). Capturing the correlation time series was done with different window sizes because smaller windows had a better temporal resolution while larger windows had stronger correlations overall; a result of the Heisenberg uncertainty principle (e.g., Grinsted et al., 2004; Hoitink & Jay, 2016). Correlations with the AMO had 15 and 25‐year filters and with Niño 3.4 (ENSO) had 3 and 10‐year filters. To analyze the data for this study and generate figures, all processing was completed with Matlab™.

## 3 Results

### 3.1 Coastal Discharge Event Frequency

**Figure 3:**
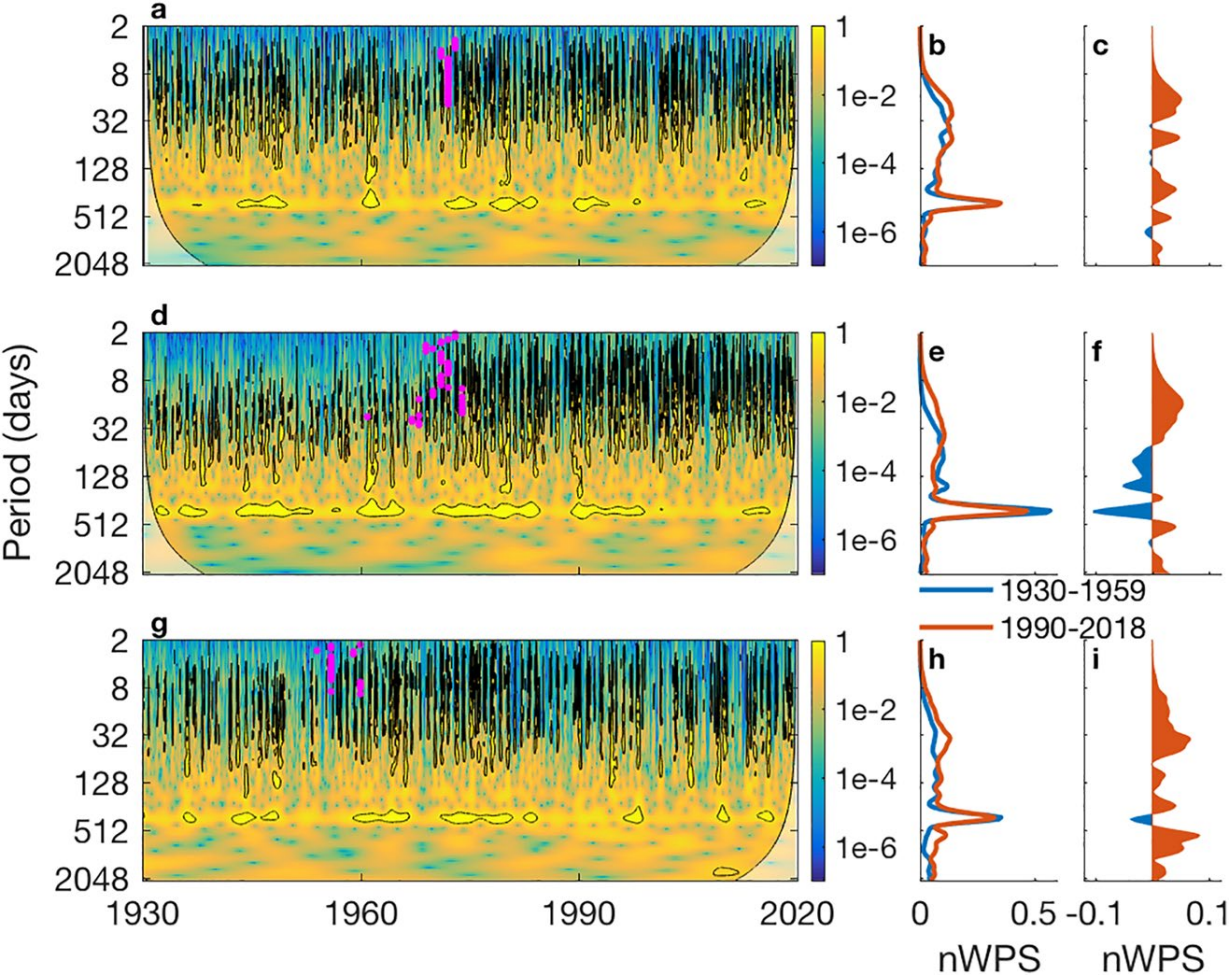
Patterns in coastal discharge energy. Wavelet power spectrum of discharge covering over 90 years for the (a) Pascagoula, (d) Tombigbee‐Alabama, and (g) Apalachicola river systems. The global wavelet power spectrum is shown for 1930–1959 and 1990–2019 (b, e, h) as well as their differences (c, f, i). All wavelet power spectrum values were normalized to reduce white and red noise by overall discharge variance and period, respectively (nWPS). The coloration in panels (a, d, g) show the nWPS as function of time (*x*‐axis) and period (*y*‐axis). Significant areas are outlined (95% CI; black line) and regions with potential edge effects in the lower corners are delineated and lightened. Horizontal bands of high wavelet power spectrum indicate a consistent frequency through time where as vertical banding represents a large event that spanned many periods. Identified change points (*p* < 0.05) for a given period are indicated (magenta dots) in panel (a, d and g)

River discharges from inland precipitation annually ranged multiple orders of magnitude as it entered fluvial‐marine transitions. Average discharges for the Pascagoula, Tom‐Al, and Apalachicola watersheds were 404, 1,866, and 675 m^3^ s^−1^, respectively. To quantify the variability in these discharge time series, continuous wavelet transformations were conducted to identify potential changes over time. For all watersheds, the strongest period was 365 days, capturing an annual pattern consistent with the seasonal pattern of wet winters and dry summers (Figures 3a, 3d and 3g). In addition, there were significant vertical bands with periods ranging 4–128 days, capturing numerous discharge events. There is a clear shift over time in the discharge characteristics in all three systems with increased significant energy in the short periods (4–64 days). To more clearly quantify the energy (i.e., variance) shifts of discharge across periods, global wavelet power spectrums of the first and last 30‐year epochs (1930–1959 and 1990–2019, respectively) were produced (Figures 3b, 3e and 3h). As discussed below (Section 3.3) these periods of time conveniently coincide with warm AMO phases, limiting potential variance changes associated with climate mode shifts. As indicated by the wavelet power spectrum, the global power spectrum showed increases in shorter periods (8–32 days) and even declined at longer periods, revealing a shift to higher frequencies of discharge (Figures 3c, 3f and 3i).

This clear change in frequency/period distribution between the beginning and end epochs suggests a regime shift at some point in the 90‐year time period. To identify when discharge frequency increased, the number of significant events per year at each period (along the *y*‐axis of Figures 3a, 3d and 3g) was tested for changes through time. The number of high frequency‐short period events significantly increased in all three watersheds between the late 1950s and early 1970s (pink dots; *p*‐value < 0.1). The change was most clearly observed in the Tom‐Al, where 8‐day events increased from 0.1 to 4.1 events per year, highlighting an overall shift in all the watersheds to higher frequency flow regimes without modifying discharge volumes.

**Figure 4:**
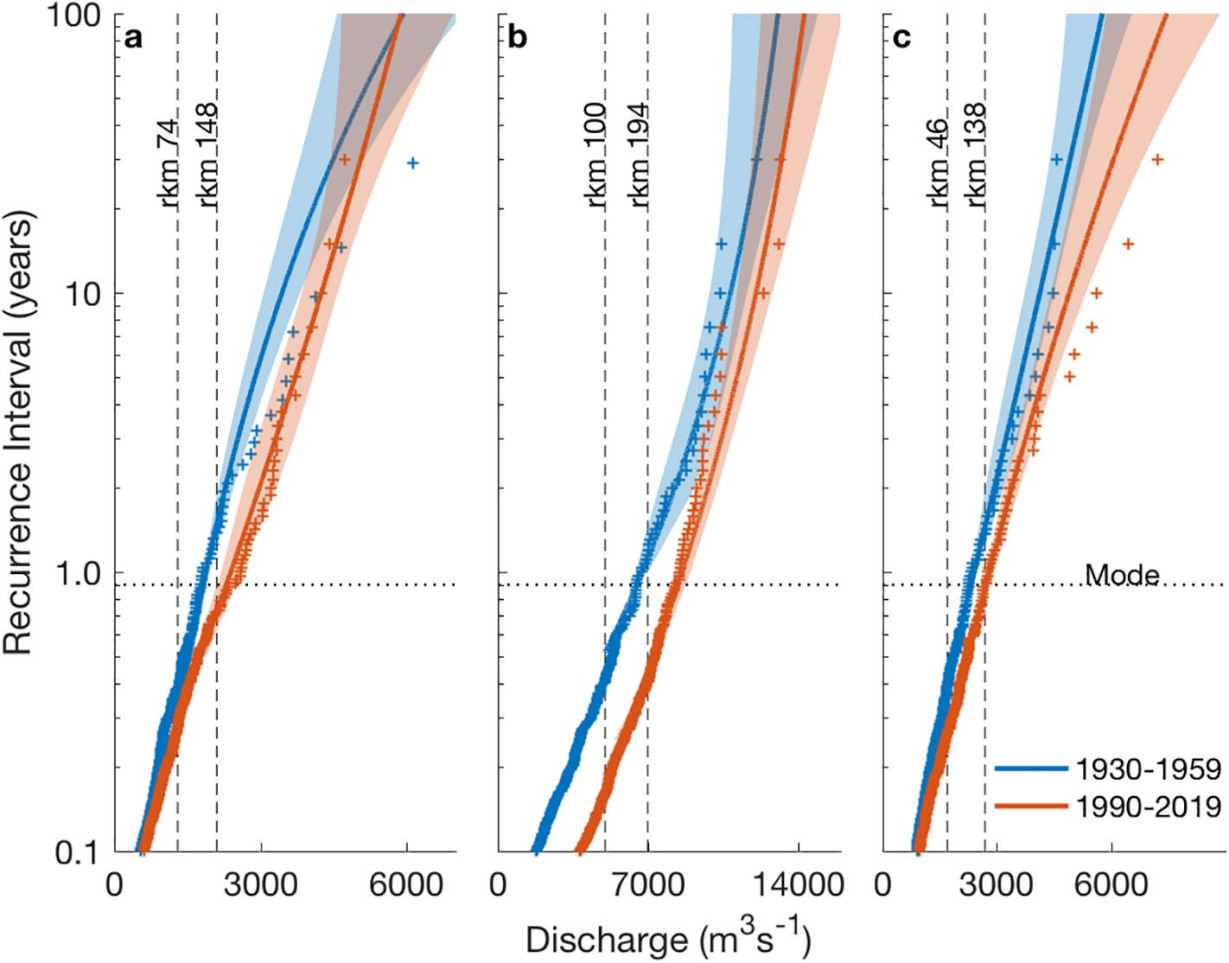
Recurrence intervals of river events. The distribution of discharge event recurrence intervals are compared for two periods based on a Generalized Pareto Distribution (GPD, lines) with a 95% confidence interval (shading) for the (a) Pascagoula, (b) Tombigbee‐Alabama, and (c) Apalachicola. For validation, a courser rank order distribution of every event is shown (“+”). In each watershed, bankfull discharge (vertical dotted lines) is shown for a site normally within the tidal reach and a site normally landward of tidal influence. Sites are labeled by river kilometers (rkm; 1 rkm = 1 km) inland from the Gulf of Mexico (Figure 2). The dotted horizontal line (0.9 years^‐1^) shows the mode bankfull discharge recurrence interval from 51 inland rivers (Williams, 1978).

Given these changes in discharge characteristics, it is natural to explore potential implications on coastal river flooding events. To better understand such impacts, recurrence intervals were calculated for the same 30‐year periods as the global wavelet spectrums in a second statistical analysis (Figure 4). The recurrences of flooding were evaluated by fitting Generalized Pareto Distributions (GPD, lines) to partial duration series (i.e., all events, not exclusively the largest annual events) to produce expected distributions of discharge events. The distributions from the two 30‐year periods were compared for differences in the frequency of flood stage conditions within each system at two different locations (i.e., bankfull discharge; vertical lines). The GPDs were generally consistent with the courser rank order event distributions (“+”), particularly near bankfull discharge, adding confidence to the patterns observed in the GPDs. In all watersheds, the two methods show recurrence intervals scaled with event size and matched well for small events (≤2 years^−1^) when the GPD differences were independent (95% CI). For all systems and locations, bankfull discharge in 1930–1959 and 1990–2019 averaged 0.88 and 0.46 years^‐1^, respectively, almost doubling the number of floods. For large events, this trend continued but was not statistically significant, with the magnitude of 1930–1959 10‐year floods approximately equaling the 1990–2019 5‐year floods. Flooding recurrence also changed spatially. In all rivers, bankfull discharge was much lower downstream (40%–62%), indicating the tidal reaches located along the fluvial‐marine transition flooded at a lower discharge than reaches further inland (Figure 4). Inland flooding recurrence intervals ranged 0.5–1.5 years^‐1^, values characteristic of rivers (Williams, 1978) where as tidal reaches had flood recurrences that ranged 0.25–0.4 years^‐1^, with the 1990–2019 Tom‐Al a bit lower at 0.19 years^‐1^. Together, averaging all systems and both time periods, tidal reaches flooded 3.2 times more often than reaches further inland. In summary, the frequency of river flooding changed temporally and spatially, doubling through time and tripling in a seaward direction.

### 3.2 Watershed Changes Through Time

**Figure 5:**
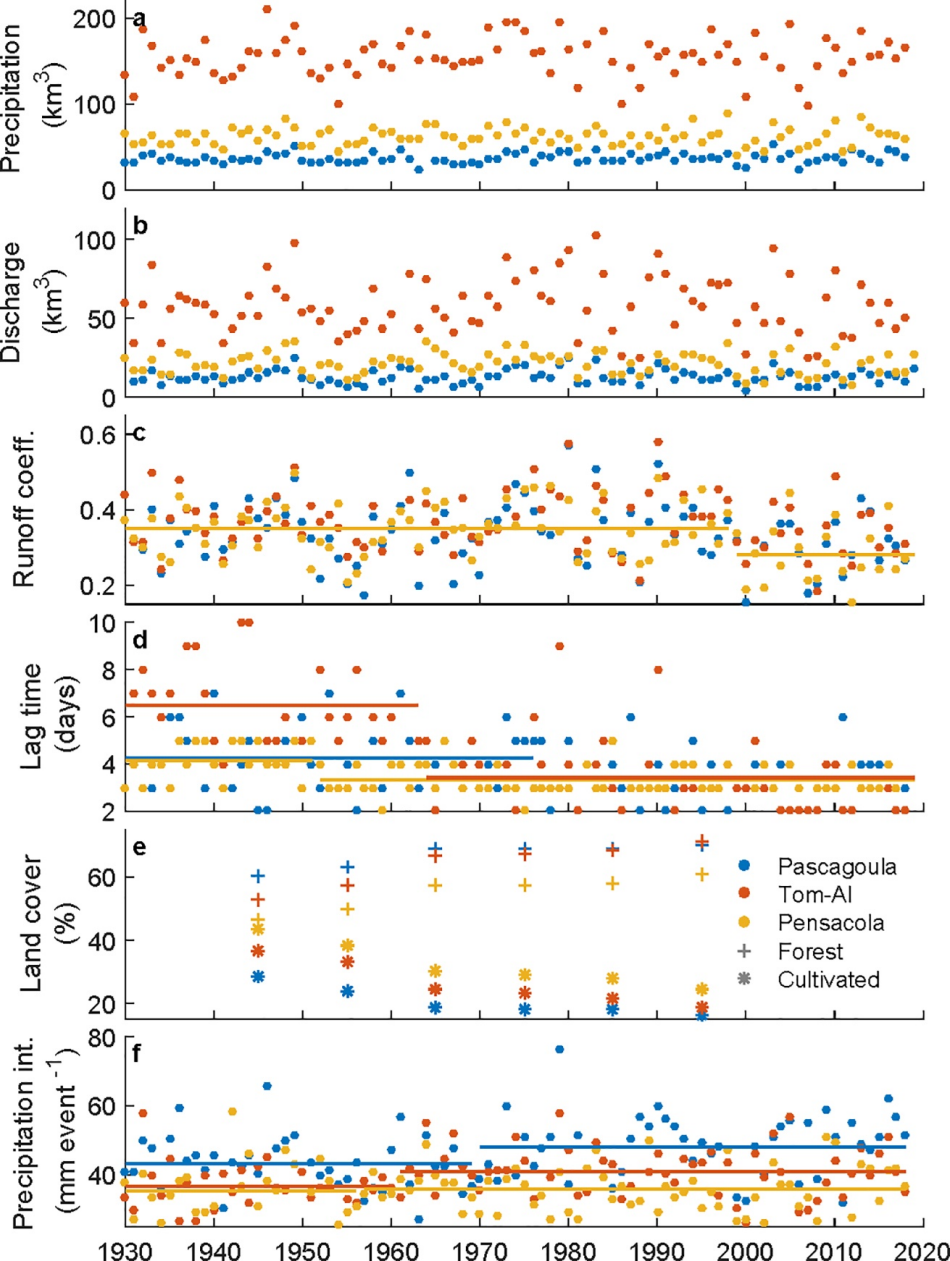
Watershed interanual variability. Time series of factors potentially affecting coastal river discharge including: (a) Precipitation, (b) Discharge, (c) Runoff coefficent, (d) Precipitation‐discharge lag time, (e) Land cover, and (f) Precipitation intensity (82nd percentile of events). Annual values (dots) are shown in panels (a, b, c, d, and f) while decadal averages (+, *) are shown in panel (e) If significant changes were detected (a–e: *p*‐value < 0.05, (f) *p*‐value < 0.1), means before and after the change point are shown (lines).

To identify why coastal river flooding frequency suddenly increased through time, watershed hydrology was examined for changes in precipitation, discharge, and land cover. In each watershed, the annual volume of precipitation entering and the volume of discharge exiting scaled with watershed size (Figures 5a and 5b). Through time no volume changes or linear trends were detected (*p* > 0.05). Comparing these volumes, the runoff coefficient had a wide range (0.16–0.63) and averaged 0.34–0.37 for all watersheds, all within commonly observed values (Figure 5c; e.g., Gericke & Smithers, 2014). One change was detected in the Apalachicola in 2000, a 25% drop indicating a relative decrease in discharge (0.35–0.28; *p* = 0.03). Comparing the timing of precipitation and discharge with an annually calculated lag time of events, discharge events lagged precipitation by 2–10 days and were longest for the largest watershed, the Tom‐Al (Figure 5d). In all watersheds, lag times significantly changed, decreasing more than 25% (*p* < 0.01). These regime shifts occurred in the mid 20th century and were largest in the Tom‐Al, averaging 6.5 days before the 1960s and 3.6 days after. The change in lag time likely played a role increasing discharge frequency even though there were no increases in the volume of precipitation or discharge.

To identify why lag times decreased, we tested land cover and precipitation intensity for changes, factors known to effect lag time (Gericke & Smithers, 2014). All watersheds were predominantly forested with a smaller cultivated portion and few developed regions (Figure 5e and Figure S2 in Supporting Information S1). From the 1940s to 1990s, approximately half the cultivated area was converted to forest, accounting for 61%–72% of the total area by the 1990s. Developed area also increased with linear trends that generally followed other regional metrics for development (e.g., road length, human population; Figure S3 in Supporting Information S1) and reached 1.2%–3.7% of the total area. The increase in forest area was 7–19 fold more than the increase in developed area, suggesting the dominant land cover change was reforestation, which should have lengthened the lag time. For precipitation intensity, the number of events in each watershed ranged between 48 and 70 per year (averaged 57–59 events per year; Figure S4 in Supporting Information S1). Events were unevenly distributed with quantile divisions of the annual volume (i.e., 0.25, 0.5, and 0.75) falling approximately at event percentiles of 64, 82, and 93, respectively, indicating most annual precipitation came in large events. Through time, the event size of most percentiles increased, indicating precipitation intensified. The largest increases were focused around the 3rd quantile of annual volume (>10%), with significant changes (*p* < 0.05) in the 82nd percentile for the Pascagoula (1972) and Tom‐Al (1961) and the 93rd percentile for the Apalachicola (1957; Figure 5f and Figure S4 in Supporting Information S1). The overall precipitation volume didn't increase because the smallest events became smaller, the number of annual events decreased through time (only significant in Pascagoula, *p* = 0.04), and the largest annual events had weak inconsistent trends; an increase in the Pascagoula and decrease in the Tom‐Al. While the timing of precipitation intensification was not concurrent, in each watershed, significant changes in precipitation intensity and lag time were within a few years of each other as well as observed changes in flooding frequency (i.e., expected range with a Pettitt Test; Figures 3 and 5).

### 3.3 Low Frequency Shift From Climate Oscillations

The precipitation intensification suggests climate may have increased the frequency of coastal river flooding. To identify the influence of climate patterns, discharge and precipitation monthly averages were tested for correlation with 14 climate indices (Table S2 in Supporting Information S1). Discharge generally had stronger correlations than precipitation. The only significant relationships with precipitation and discharge, where both were positive or negative, was the AMO and ENSO (*p* < 0.1). Importantly, the AMO and the ENSO, captured using the Niño 3.4 index, were uncorrelated (*r* = 0.02, *p* = 0.23) with periodicities that differed by approximately an order of magnitude (∼60 and ∼3–12 years, respectively; Table S3 in Supporting Information S1), indicating the affects of each oscillation could be separately detected.

**Figure 6:**
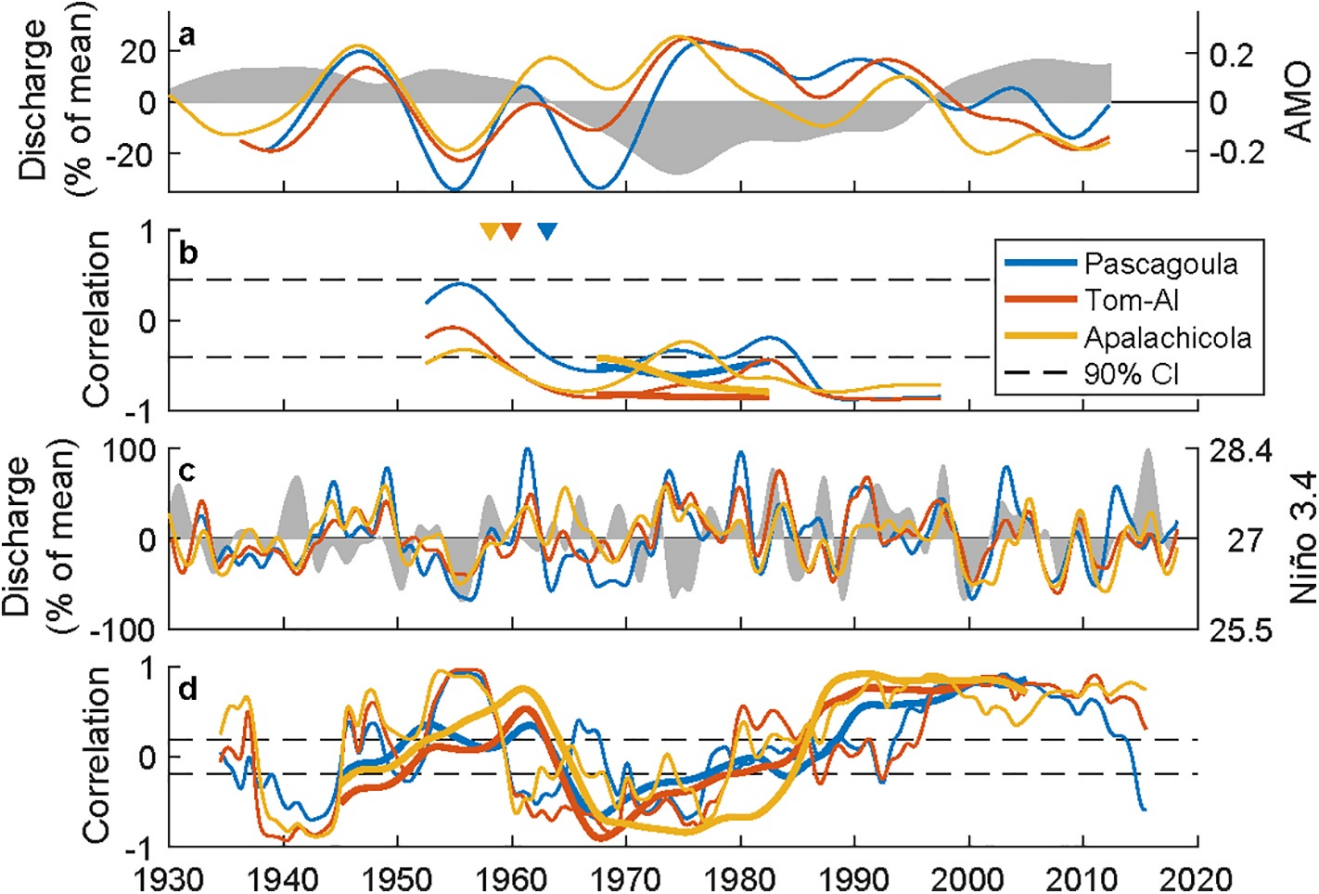
Climate oscillation‐discharge correlation. Time series of panel (a) very low frequency (15‐year filter window) normalized discharge and the AMO (gray shading), (b) Moving correlation between the AMO and discharge, (c) Low frequency (3‐year filter window) normalized discharge and Niño 3.4 (gray shading), and (d) Moving correlation between Niño 3.4 and discharge. In panel (b and d), the thin lines have confidence intervals and correspond to the thin lines and filtering of panels (a and c), respectively, while the bold lines capture lower frequencies with 25 and 10‐year filter windows, respectively. In panel (b), triangles at the top of the plot indicate when the AMO‐discharge correlation became significant with a 15‐year filter window.

To identify changes in the influence of the AMO and ENSO on flooding events, moving correlations with filtered discharge time series were conducted (e.g., Enfield et al., 2001). Focusing first on the lowest frequency pattern, there was a general out of phase relationship between discharge and the AMO, particularly in the latter two‐thirds of the time series (Figure 6a). This is confirmed in the correlation analysis where the two time series are negatively correlated over this entire interval for both low and very low frequency filtering of the time series (Figure 6b). At higher frequency, the relationship between discharge and ENSO becomes more complicated with periods of significant positive and negative correlations (Figures 6c and 6d). Interestingly, the change in the sign of the ENSO‐discharge correlation followed the pattern of the AMO for most of the time series. During the 1950s to early 1960s and after the mid‐1990s when the AMO was positive (i.e., a warm phase), the ENSO‐discharge correlation was generally positive. In contrast, when the AMO phase was negative (late 1960–1970s), the ENSO‐discharge correlation became negative. The correlation broke down in the 1980s when the cool phase (negative values) of the AMO was waning and weak.

For precipitation, the time series began a few decades earlier and produced similar correlations (Figure S5 in Supporting Information S1). A couple differences were observed, including positive AMO‐precipitation correlations in the early 20th century and noisier Niño 3.4‐precipitation correlations. Regardless of these differences, in each watershed, the time the AMO began to have strong negative correlations with precipitation and discharge closely reflected when precipitation intensified, lag time decreased, and the frequency of coastal river flooding increased.

### 3.4 Individual Events

The impacts of the AMO and ENSO related climate variability on precipitation intensification and subsequent flooding in the fluvial‐marine transition can be more clearly observed through examining individual river and compound fluvial‐marine events. Events in the Tom‐Al fluvial‐marine transition are used because it is a representative system with single strand rivers, a long delta, and a large bay (Figure 1a). Focusing first on the impacts of river events on coastal flooding, March 2010 is highlighted (Figure 1b). This was one of the wettest months in the last 30‐year epoch with four precipitation events (March 2, March 10–11, March 22, and March 25). All events produced river waves (i.e., sequential water level peaks along the fluvial‐marine transition) that became smaller and occurred later in a seaward direction, attenuating and slowing as the waves propagated (∼130– ∼60 cm s^‐1^). Only the extreme event caused flooding (March 16–24), but instead of it having the shortest lag time, waters rose slower and fell quicker near peak water level (i.e., negative skew), making the lag time ∼3 days longer than the small events. Thus, these two responses (i.e., lag time and discharge event magnitude) to precipitation intensity are in and of themselves critical determinants of coastal flooding, but they also have implications for compound events.

Compound events are highlighted by the passage of hurricanes Katrina and Rita in 2005. These tropical cyclones created both river and storm surge waves (Figure 1c). The storm surges exceeded a meter at the mouth of the system (rkm 0), more than three times the tidal amplitude, and propagated landward. Successive peaks are clearly visible at 0 rkm and 100 rkm prior the initiation of the river wave and became convolved with the river wave at rkm 194 (i.e., evidence of a compound event). Thus, the timing (and hence location) of the compound event as well as the magnitude of the river wave that is involved in the compound event is, in part, determined by the intensity of the precipitation which set the precipitation‐discharge lag time and the initial river wave magnitude.

It is important to note that these August and September hurricanes had similar precipitation to March 10–11, 2010 and compounded with storm surge yet did not flood the fluvial‐marine transition like the March 2010 coastal river flooding event. This was consistent with long‐term observations of 127 delta‐flooding events (1985–2019), none of which were compound events during peak hurricane season (August–October). Despite the possible flooding from storm surge at the seaward end of the fluvial‐marine transition, most flooding along the fluvial‐marine transition was a direct result of high river discharge.

## 4 Discussion

### 4.1 Precipitation Intensifications Impact River Dynamics

Long‐term observations captured increases in coastal river flooding frequency, which resulted from precipitation intensification decreasing the precipitation‐discharge lag time. In each watershed, there were near concurrent changes in the time of precipitation intensification (Figure 5f), the decrease in precipitation‐discharge lag time (Figure 5d), and the increase in discharge frequency (Figure 3). This suggests the larger precipitation events more quickly saturated the earth surface, causing runoff to move faster into streams and the fluvial‐marine transition. Instead of long lag times in watersheds temporally filtering events, resulting in a season of high discharge, each intense precipitation event formed a pulse of discharge, resulting in a higher frequency and magnitude of flooding events. Similar affects of precipitation on inland flooding are observed in individual events (e.g., Gericke & Smithers, 2014; Turner‐Gillespie et al., 2003) and through time (e.g., Mallakpour & Villarini, 2015), which are shown here to affect coastal river flooding.

The results of this coastal river flooding study may be limited by our selection of watersheds and processes impacting watershed hydrology in a geographicly limited data set. The selected watersheds were not dry, continental sized (e.g., Mississippi, Nile), or urban, and so extending the findings of this study may merit additional investigation to address these other environments. One study on urbanization shows Houston, Texas has escalated the effects of precipitation intensification in their small watershed, magnifying an extreme flood from Hurricane Harvey (Sebastian et al., 2019). Scaling up direct human impacts to large watersheds can be misleading (Wohl, 2019) and is a knowledge gap not filled by this study as the developed regions were relatively small (<3.7%, compard to Houston at 79%; Sebastian et al., 2019), suggesting urban effects may be present but were minimal. The predominant land cover change was reforestation, a process shown to reduce runoff (Hopkinson & Vallino, 1995; Trimble & Weirich, 1987). Other processes impacting watershed hydrology may have also lessened the effects of intensifying precipitation, such as waterway projects, like the Tennessee‐Tombigbee Waterway built in the 1980s, and water loss, like the 25% drop in the Apalachicola runoff coefficient. The Apalachicola water loss also corresponds to a lack of discharge events in the 21st century when precipitation was low (Figures 3g and 5a), suggesting it may have been caused by increased regulation that is part of an ongoing interstate dispute between Florida, Georgia and Alabama. Despite these human impacts, the largest changes in flooding frequency and lag time still occurred in the mid 20th century when precipitation intensified.

The critical link between precipitation intensification and the higher frequency of coastal river flooding was the precipitation‐discharge lag time, or speed of an event. The observed intensification of most precipitation without a significant increase in the largest annual precipitation event is consistent with precipitation changes in the central United States, where river flood frequency also increased without a large increase in event magnitude (Mallakpour & Villarini, 2015). This may be unexpected because precipitation event magnitude scales with overland runoff speed (McCuen & Spiess, 1995), controlling the flow of water and overall flooding. However, in rivers, magnitude and event speed can be dynamic. If wide floodplains are present, river wave speed peaks when water levels slightly exceed bankfull conditions and can decrease as events become larger (Knight & Shiono, 1996; Wolff & Burges, 1994). This occurs because floodplains temporarily store water, causing downstream attenuation of flooding, delayed peak water levels, and longer lag times (Dykstra & Dzwonkowski, 2020a; Fleischmann et al., 2016). These hydraulics are occasionally observed in inland rivers (e.g., Lininger & Latrubesse, 2016; Turner‐Gillespie et al., 2003), and because floodplains generally broaden in a downstream direction, they are more likely to occur in coastal river floods (Dykstra & Dzwonkowski, 2020a; Fleischmann et al., 2016). For example, of all the observed Tom‐Al discharge events in Figure 1, the flood had the flattest, most negatively skewed hydrograph (i.e., leftward) and longest lag time. As a result of flooding, the shortest lag times are most likely to be caused by a critical range of large precipitation events that are not large enough to greatly exceed bankfull discharge. As climates change, overall lag times should decrease the most if small and medium events become larger while extreme events become smaller. This could greatly increase the frequency of flooding regardless of extreme event magnitude, as observed in this study and the central United States (Mallakpour & Villarini, 2015).

### 4.2 River Responses Impact Coastal Flooding Dynamics

Not only did flooding increase through time, it also increased in a downstream direction. Spatial changes have also been observed on the coastal plain of North Carolina (Sweet & Geratz, 2003), where one of the lowest recorded flooding intervals of 0.19 year^−1^ are consistent with the lowest interval observed in the Tom‐Al (Figure 4b). Similar trends are empirically observed on inland rivers showing recurrence intervals decrease with decreasing river slope, and are attributed to flow deceleration and relatively low riverbanks (Copeland et al., 2005; Williams, 1978). Because slope generally decreases downstream, approaching zero near the sea (i.e., backwater environments), the highest frequency of river flooding should be in the fluvial‐marine transition, as observed in this study.

**Figure 7:**
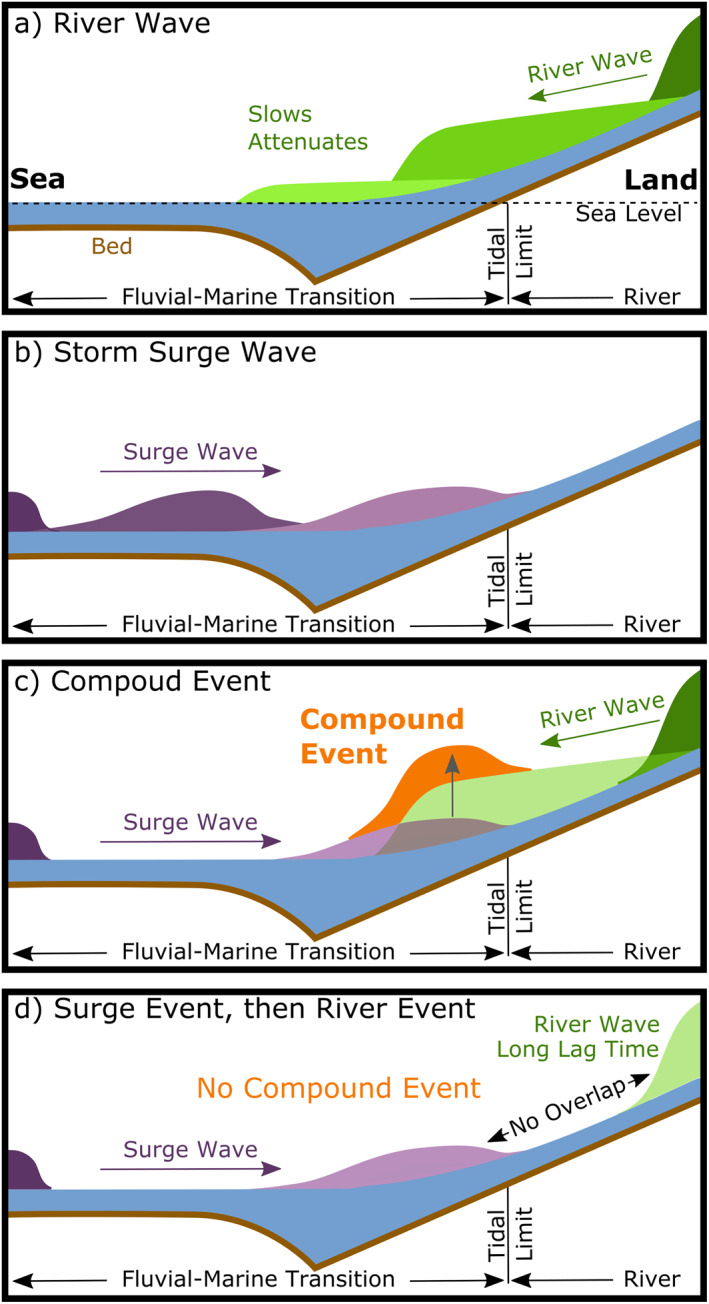
Conceptual diagram of compound river‐storm surge events in the fluvial‐marine transition. The longitudinal profiles show (a) a river event (green) and (b) a storm surge event (purple) at three different times, capturing the events propagating as long waves and (c), (d) scenarios of how the waves can interact. (c) If the river and surge waves coincide in the same space at the same time, they form a compound event where water levels are higher than either event alone. (d) If the river wave is delayed (e.g., long precipitation‐discharge lag time, flooding), the surge wave may attenuate before the river wave reaches the surge wave. In this scenario, the surge event is followed by a river event and no compound event occurs.

Along the fluvial‐marine transition, river event (i.e., river wave) water levels were additionally elevated by storm surge, forming compound events. Compounding occurred when intense precipitation created discharge events with short lag times (Figure 7c). If these discharge events had flooded, like March 2010 when antecedent discharge was already elevated, flooding could have increased the lag time due to overbanking (Dykstra & Dzwonkowski, 2020a), reducing the likelihood of compounding (e.g., Figure 7d). This overbanking transition to slower river waves may explain why intense precipitation during peak hurricane season only formed non‐flooding compound events.

The compound events occurred when river and marine waves simultaneously propagated through the same region and may be the first observations of compound river‐marine events to be described as compounding waves (Figure 7). Capturing the waves in the same region allows for a simple comparison, which shows river waves had larger amplitudes entering the fluvial‐marine transition, more attenuation, and slower propagation (Figures 7a and 7b). These differences are attributed to their momentum in the shallow sloping fluvial‐marine transition with river waves loosing momentum and surge waves retaining momentum (Dykstra & Dzwonkowski, 2020a). The difference in celerity makes compound events most likely to occur on the inland reaches of the fluvial‐marine transition, near the tidal limit, where water levels would also be highest due to less attenuation of the river wave (Figures 1c and 7c). Other observations support this spatial variability of compound events, showing high risk for cities located near tidal limits (e.g., Washington, DC) and lower risk for cities directly on the coast (e.g., San Francisco; Moftakhari et al., 2017).

### 4.3 Climate Change and Climate Modal Variability

The observed shifts in all three systems appear to be connected to climate patterns. While the correlations observed in the climate modal analysis do not necessarily imply causation, coupled atmospheric‐oceanic processes do provide mechanistic connections. For North America, Atlantic sea surface temperature, which the AMO measures, has a particularly important affect on climate through intensifying and weakening the North Atlantic Subtropical High (NASH), which modulates the Pacific‐North American Jet Stream and atmospheric circulation (Hu & Feng, 2012). Following a southward shift of the NASH, more moist air began advecting from the Gulf of Mexico in the 1950s, increasing the number of frontal storms and precipitation intensity in the central and southern United States (Bishop et al., 2019). Our results suggest this change in climate provided a mechanistic connection through which the AMO began strongly modulating precipitation and discharge. The regime changes of each system occurring at different times were likely caused by their spatial proximity to the NASH, first affecting the Apalachicola in the east and the Pascagoula last in the west.

The AMO‐NASH patterns also have teleconnections with ENSO in North America. The Niño 3.4‐precipitation correlation is negative in the Great Lakes Region and positive in the Florida Peninsula resulting in a neutral transition region across the eastern United States. The specific spatial location and extent of this neutral region is influenced by the strength of the NASH, which connects the influence of ENSO to the longer scale phase of the AMO (Enfield et al., 2001; Hu & Feng, 2012). Here, our study region is nearest Florida and discharge is commonly assumed to have a strong positive correlation with Niño 3.4 (e.g., Chigbu et al., 2004; Gomez et al., 2019). However, using nearly a century of observations, the results showed both positive and negative correlations with significantly higher discharge during warm AMO El Niño and cool AMO La Nina years.

### 4.4 Broader Implications

#### 4.4.1 Precipitation Intensification May Affect Many Fluvial‐Marine Transitions

By linking climate change and variability to watershed processes and coastal flooding, the findings of this regional study provide a broad context applicable to other settings. Due to the complexity of watershed hydrology, other studies examining the role of precipitation intensification on coastal flooding have not addressed watershed dynamics and use proxies to estimate discharge (e.g., runoff coefficient; e.g., Bevacqua et al., 2018, 2020; Wahl et al., 2015). Their results suggest the coastal threat from precipitation intensification is niche to compound events in the fluvial‐marine transition of very small watersheds (<5,000 km^2^). In contrast, our results show precipitation intensification, even far from the coast, can shorten the lag time and increase the frequency of coastal river flooding in the fluvial‐marine transition of medium and large watersheds. For larger watersheds, proxy methods may not be appropriate for the nonlinear behavior caused by overbank flooding conditions (i.e., lag time, peak discharge, time of concentration). This also raises questions for the common practice of predicting flood risk from the largest annual precipitation and discharge events, which may be the slowest and most attenuated coastal river floods.

#### 4.4.2 Accounting for River Dynamics in Coastal Flooding

For coastal hydrology, some complex marine influences along the fluvial‐marine transition may also be averaged out, but should not be ignored. For example, the higher frequency and magnitude of coastal river flooding, compared to inland flooding, will likely increase through time as sea levels continue to rise, amplify tides (Devlin et al., 2017) and outpacing most natural levee formation (e.g., Giosan et al., 2014), further escalating the already higher risk of coastal river flooding. Additionally, the greatest likelihood of compound flooding near the tidal limit suggests integrating fluvial hydrology approaches, like observing the river wave here, is critical for determining flooding risk. At this point, few observations of compound floods are even attempted near the tidal limit. Many studies attempt to capture the variability of compound flooding in a system with one gage in tidally dominant regions (e.g., Hendry et al., 2019; Ward et al., 2018), missing the spatial variability of fluvial‐marine transitions, a risk that may threaten communities located at the head of an estuary or along a coastal river.

One of the most important factors for coastal flooding is watershed dynamics, of which size plays a critical role. In small watersheds, the largest fastest moving events form flash floods (Gericke & Smithers, 2014), but as floodplain size increases with watershed size in alluvial systems (e.g., Fleischmann et al., 2016), the largest discharge events may become too slow to compound with storm surge (Figure 7b). The compound floods are caused by small river events because they travel fastest. While compound events from a single low‐pressure system are shown to impact coastal areas, our regional results demonstrate such events in large watersheds are of limited importance, consistent with previous work (Bevacqua et al., 2020; Hendry et al., 2019). Because river events are commonly higher than surge events along most of a fluvial‐marine transition, the largest flood hazard may be solely from river discharge. However, if human impacts have restricted channels from their floodplains (e.g., levees, polders; Helaire et al., 2020), a common practice for inland flood protection that increases the speed of discharge events (Hopkinson & Vallino, 1995), the risk of compound floods could escalate with development and watershed size.

#### 4.4.3 Reevaluating Climate Affects on Coastal Flooding

River flooding and discharge affected by AMO‐ENSO relationships similar to this study have been observed outside North America (Aalto et al., 2003; Barichivich et al., 2018). Similar concurrent changes in the Amazon Basin suggest the mid 20th century reorientation of AMO‐ENSO teleconnections could have also affected the tropics (Aalto et al., 2003). These wide reaching teleconnection changes suggest a better understanding of effects from AMO‐ENSO variability may be warranted. Because of the central role of ENSO in the management of water resources, food safety, and marine ecology (Chigbu et al., 2004; Gomez et al., 2019), our results suggest ENSO related research from the current warm AMO may need to be reevaluated as the AMO becomes cool, a process likely to occur within the next decade.

In reevaluating the role of the AMO, ENSO, or other climate oscillations, a couple modifications could improve results. First, long‐term data sets are needed to capture the role of long climate periods like the AMO. For example, using data from the latter half of the 20th century, numerous studies of North American rivers that include the rivers of this study, show a near linear increase in discharge volume through time (e.g., Milliman et al., 2008), while studies with data staring in the latter 20th century show a decreasing trend. Using longer time series, our results suggest discharge volume and flooding frequency are nonlinear through time and the relationships with the NASH and the AMO have likely been overlooked or misinterpreted. Second, teleconnections and climate change affect most environmental observations (Bauer et al., 2013; Hu & Feng, 2012), indicating temporal variability in a signal should be expected. A single correlation can overlook variability or partially average out the role of a climate oscillation. Temporally flexible methods, such as windowing correlations and wavelets (Figures 3 and 6c), provided important insights and could greatly improve other long‐term analyses.

The changes in climate associated with the NASH and the AMO suggest coastal river flooding and compound fluvial‐marine events have become more frequent with precipitation intensification and will continue increasing with global warming. Global warming has already intensified precipitation across most of the tropics (Allan & Soden, 2008) and in many temperate regions, including parts of the continental United States where precipitation is expected to intensify for the entire region by 2100 (Easterling et al., 2017). Similarly, in high latitudes, runoff has increased with more precipitation coming as rain, changing the character of river flooding (Shiklomanov et al., 2007). Our regional results suggest the global intensification of precipitation may already be increasing the global frequency of river flooding and poses the greatest risk along the fluvial‐marine transition for two reasons. First, river flood frequency increased downstream, peaking in tidally influenced reaches. Second, precipitation intensification shortening lag times strongly increases the likelihood of compound events from a single low‐pressure system. Because lag time may not scale with precipitation magnitude, particularly when floods occur, calculating this risk will require predicting which precipitation events intensify (e.g., percentile, season) and how it relates to bankfull discharge. An intensification of only the most extreme precipitation could magnify large coastal river floods, and lengthen lag time, simultaneously reducing the likelihood of compound events. Overall, our findings suggest the current global precipitation intensification may have the greatest flooding consequences downstream, in coastal river floods.

## 5 Conclusions

This regional study demonstrates changes in climate and climate oscillations can increase the frequency of coastal river flooding through the intensification of inland precipitation. This intensification decreased the lag time of coastal river discharge events, an increase in transport efficiency that caused coastal river discharge to more closely reflect the higher frequency of precipitation. Shorter lag times can also increase the likelihood of compounding with storm surge, which was observed in a large watershed, far inland near the tidal limit. However, compound flood risk may be limited if extensive inland river flooding delays river flooding in the fluvial‐marine transition, making the largest flood hazard along most of the fluvial‐marine transition solely from river discharge, as demonstrated here. These hydrological patterns where modulated by changes in the regional climate that were driven by the AMO‐NASH ocean‐atmosphere teleconnections. The teleconnections caused positive and negative ENSO correlations, suggesting the wide‐ranging affects of ENSO (e.g., water management, food safety, marine ecology) may need to be reevaluated as the AMO begins its transition to a cool phase. Our findings are broadly relevant to other systems as climate change is expect to globally intensifying precipitation, suggesting the frequency of coastal river and compound fluvial‐marine flooding will increase with warming atmospheric temperatures, putting the most dense human populations and infrastructure of the world at high risk.

## Conflict of Interest

The authors declare no conflicts of interest relevant to this study.

## Supporting information

Supporting Information S1

## Data Availability

All data needed to recreate this analysis are publicly available through the NOAA (Global Historical Climatology Network: https://www.ncei.noaa.gov/products/land-based-station/global-historical-climatology-network-daily, Global Precipitation Climatology Center: https://psl.noaa.gov/data/gridded/data.gpcc.html, Tides & Currents: https://tidesandcurrents.noaa.gov/, Global Climate Observing System: https://psl.noaa.gov/gcos_wgsp/Timeseries/), the USGS (National Water Information System: https://waterdata.usgs.gov/nwis, Watershed Boundary Dataset: https://www.sciencebase.gov/catalog/item/5a1632b3e4b09fc93dd171e2, Wieczorek et al., 2019, https://www.sciencebase.gov/catalog/item/58cbeef2e4b0849ce97dcd61, Rating Curve Builder: https://waterwatch.usgs.gov/?id=mkrc), and Dykstra and Dzwonkowski (2020b): https://data.gulfresearchinitiative.org/data/R4.x260.000:0125, and are further detailed in Table S1 of the Supporting Information S1.
