# Supplementary material for: The Role of Intensifying Precipitation on Coastal River Flooding and Compound River‐Storm Surge Events, Northeast Gulf of Mexico
Source: Water Resour Res. 2021 Nov 10;57(11):e2020WR029363. doi: 10.1029/2020WR029363 (PMC9286652; doi:10.1029/2020WR029363)
Supplement: Supplementary file 1 — Supporting Information S1 [file WRCR-57-0-s001.pdf]

Water Resources Research

Supporting Information for

The Role of Intensifying Precipitation on Coastal River Flooding and Compound River-Storm Surge Events, Northeast Gulf of Mexico

S. L. Dykstra[1,2] and B. Dzwonkowski[1,2]

[1]Department of Marine Sciences, University of South Alabama, Dauphin Island Sea Lab, Dauphin Island, AL 36528.

[2]Dauphin Island Sea Lab, Dauphin Island, AL 36528.

Contents of this file

Figures S1 to S5
Tables S1 to S3

Introduction

The supporting figures and tables are described and discussed in the main text. Additional government reports were accessed for road lengths (U.S. Bureau of Public Roads 1952, 1962, U.S. Federal Highway Administration 1972, 1982, 1992, 2003, 2014, 2021) and population (U.S. Census Bureau 2021).

Supporting Figures

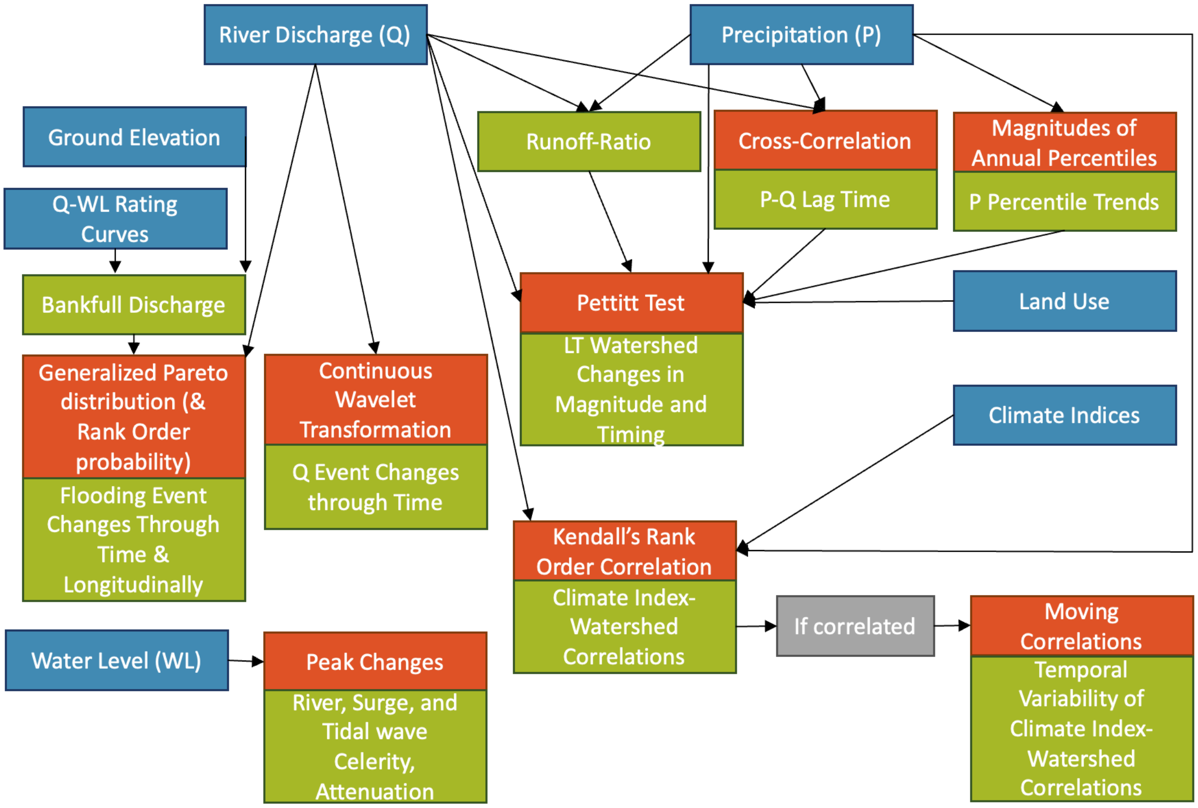

Figure S1. Flowchart of the methodology. Blue, red, and green boxes show observations/source materials, methods, and results, respectively.

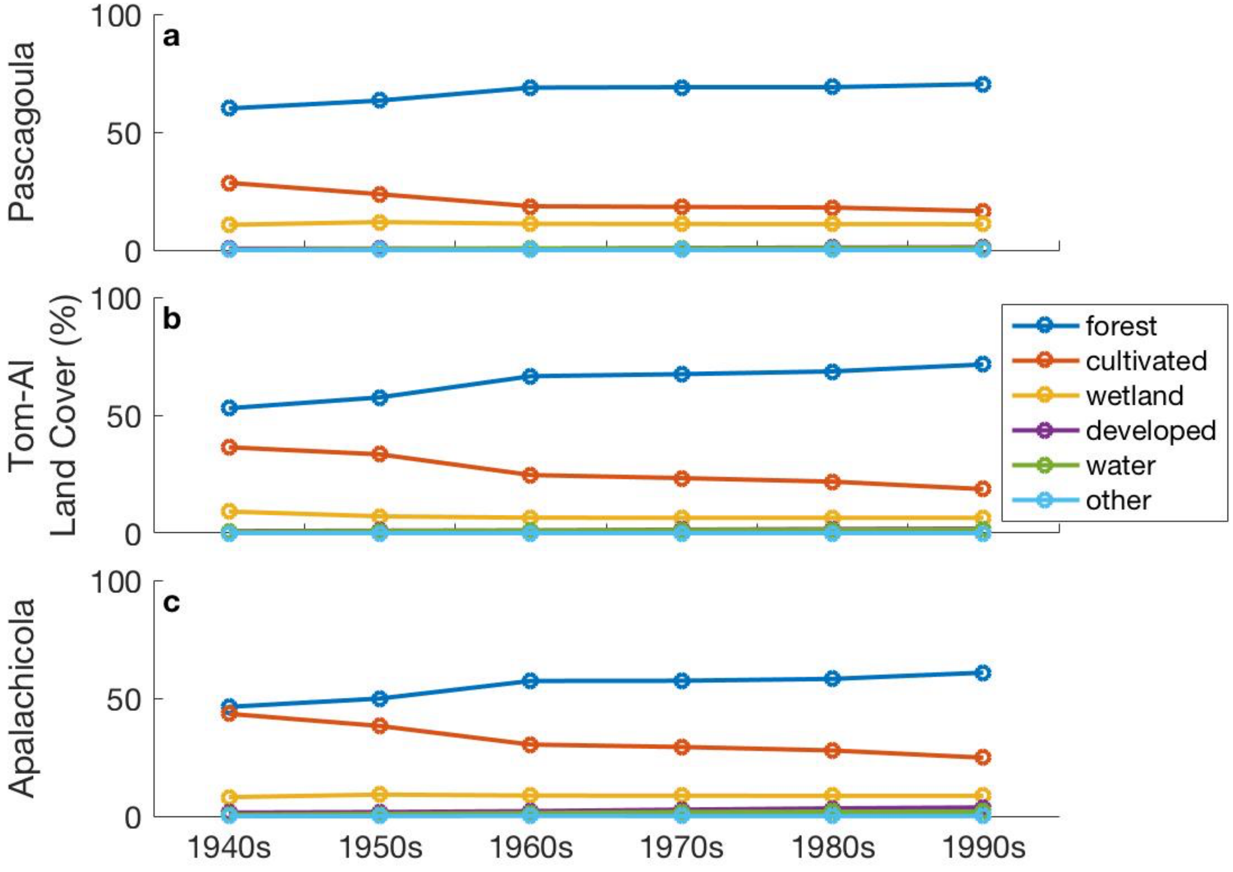

Figure S2. Land cover time series. Land cover is shown by watershed for the (**a**) Pascagoula, (**b**) Tombigbee-Alabama, and (**c**) Apalachicola.

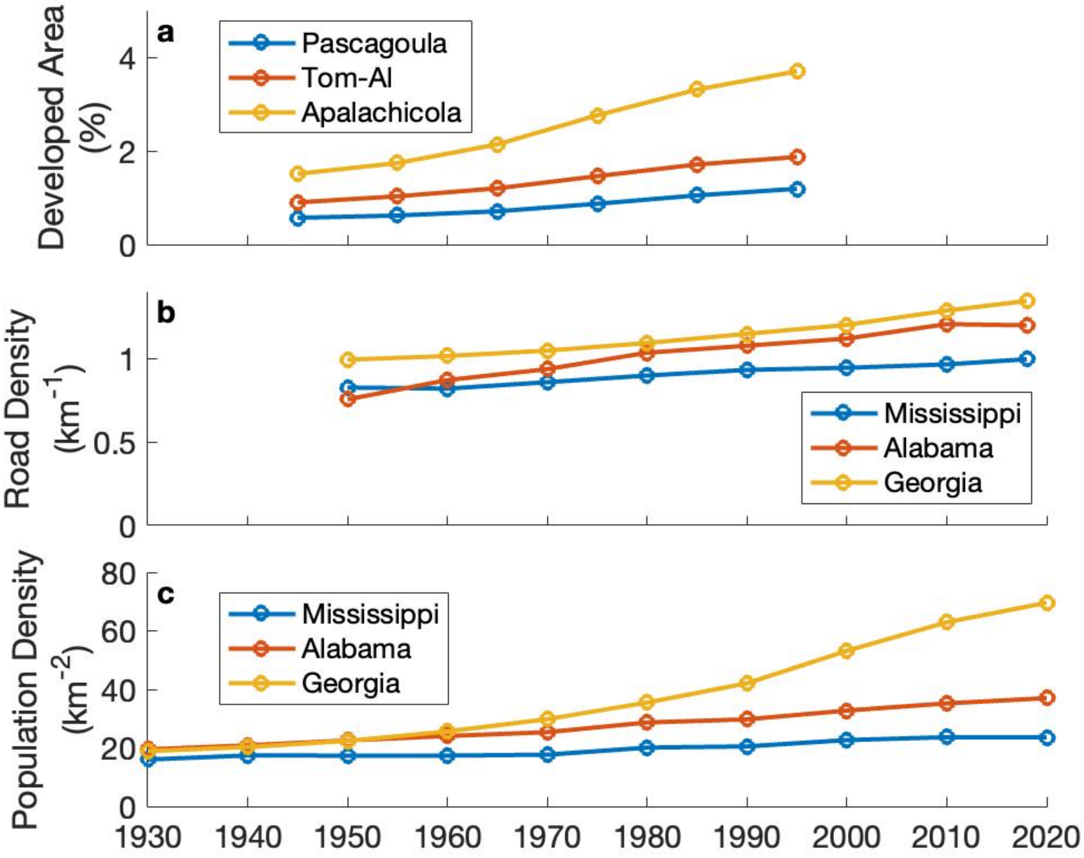

Figure S3. Human development through time. Time series of (**a**) developed area by watershed and the densities of (**b**) roads and (**c**) human population by state. (**b**) Roads commonly have ditches, which allows them to serve as an estimate to identify changes in drainage density. The Pascagoula, Tombigbee-Alabama, and Apalachicola watersheds are predominantly within the state of Mississippi, Alabama, and Georgia, respectively. (**c**) Trends in road and population densities support the modeled trends in developed area. The Georgia population trend being steeper than the Apalachicola developed area is likely a result of the metropolitan region of Atlanta, of which only part is within the Apalachicola Watershed. Note: (**a**) Because developed area is small in context of other land covers (Fig. S2), for clarity, it is also shown here.

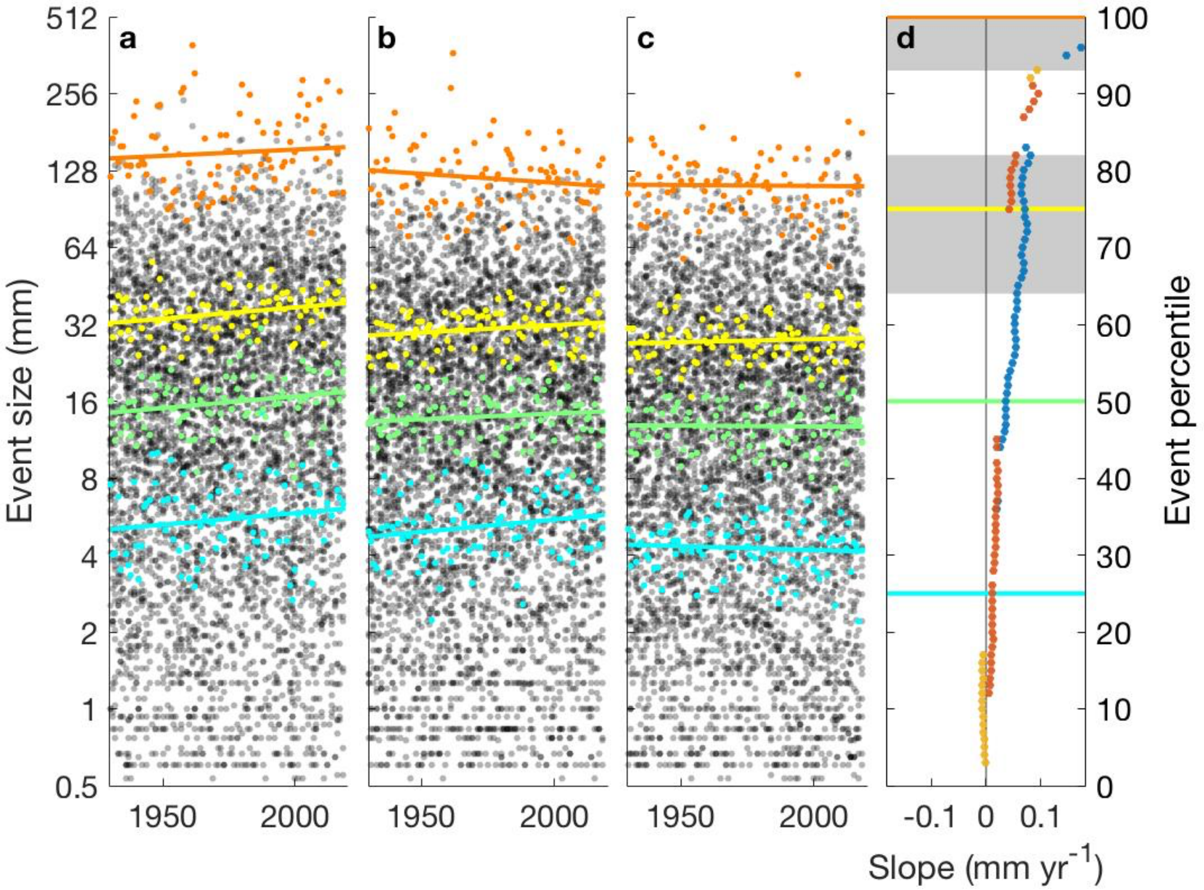

Figure S4. Precipitation event trends. Time series of precipitation event sizes (gray dots) highlighting linear trends of the 25th, 50th, 75th, and 100th percentiles (light blue, green, yellow, and orange lines, respectively) for (**a**) Pascagoula, (**b**) Tom-Al, and (**c**) Apalachicola Watersheds. (**d**) A summary of significant percentile trends ($p<0.1$) over time highlights long-term changes in system events where positive slopes indicate increasing magnitudes for Pascagoula (blue), Tom-Al (red), and Apalachicola (dark yellow) Watersheds. Highlighted event percentiles from **a**, **b**, and **c** are shown in contrast to the mean distribution of volume (1st/3rd and 2nd/4th quartiles in white and gray banding, respectively), showing half of all precipitation is represented by the 82nd percentile and came in the largest 18% of events.

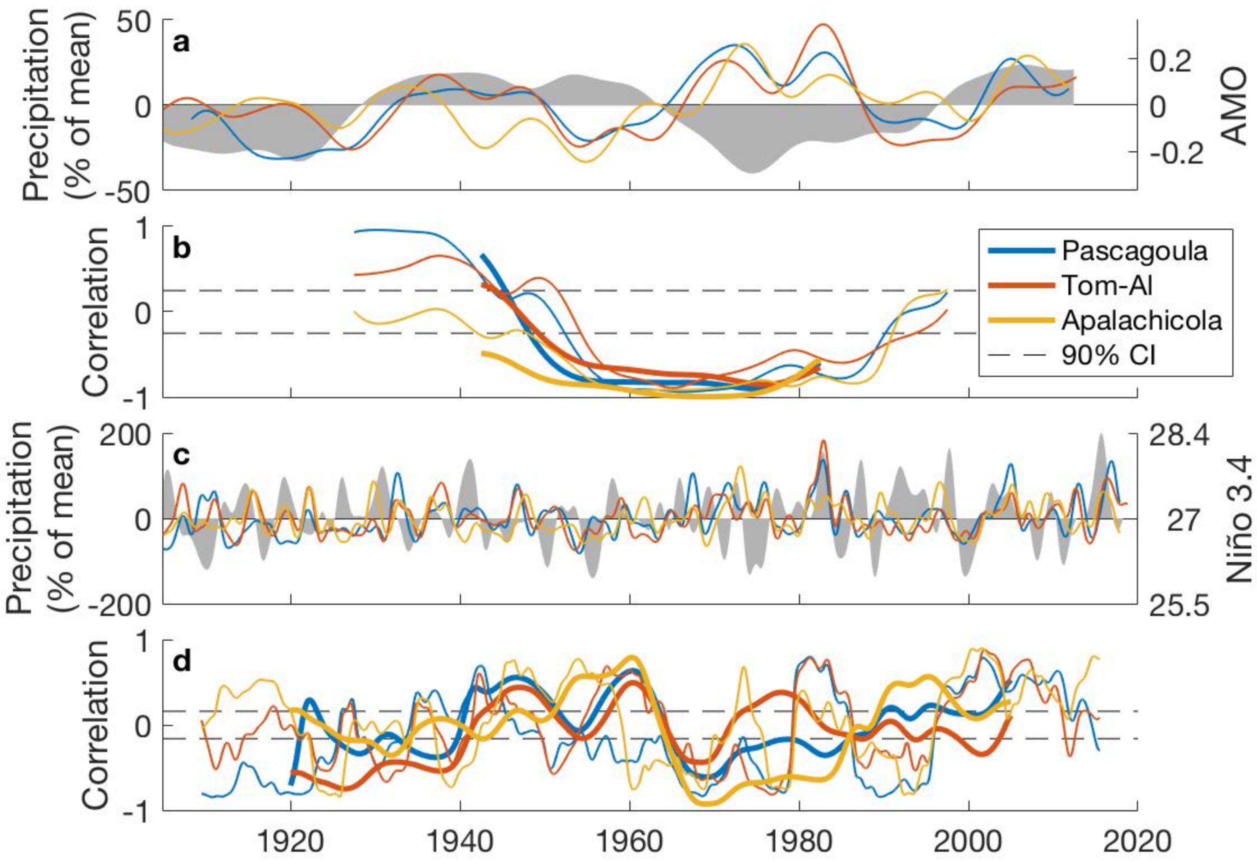

Figure S5. Climate oscillation-precipitation correlation. Time series of (**a**) very low frequency (15-year filter window) normalized precipitation and the AMO (grey shading), (**b**) moving correlation between the AMO and precipitation, (**c**) low frequency (3-year filter window) normalized precipitation and Niño 3.4 (grey shading), and (**d**) moving correlation between Niño 3.4 and precipitation. In **b** and **d**, the thin lines have confidence intervals and correspond to the thin lines and filtering of **a** and **c**, respectively, while the bold lines capture lower frequencies with 25 and 10-year filter windows, respectively.

Supporting Tables

Table S1. Data list and sources. Table shows all data used in the study and URLs for direct data access.

Data Type	Source	Name	ID	Resolution	URL
Precipitation	NOAA	Global Historical Climatology Network			ncdc.noaa.gov/ghcnd-data-access
		Aberdeen, MS US	USC00220021	Daily	ncdc.noaa.gov/cdo-web/datasets/GHCND/stations/GHCND:USC00220021/detail
		Albany 3 SE, GA US	USC00090140	Daily	ncdc.noaa.gov/cdo-web/datasets/GHCND/stations/GHCND:USC00090140/detail
		Hattiesburg, MS US	USC00223887	Daily	ncdc.noaa.gov/cdo-web/datasets/GHCND/stations/GHCND:USC00223887/detail
		Leakesville, MS US	USC00224966	Daily	ncdc.noaa.gov/cdo-web/datasets/GHCND/stations/GHCND:USC00224966/detail
		Montezuma 2 NW, GA US	USC00095979	Daily	ncdc.noaa.gov/cdo-web/datasets/GHCND/stations/GHCND:USC00095979/detail
		Selma, AL US	USC00017366	Daily	ncdc.noaa.gov/cdo-web/datasets/GHCND/stations/GHCND:USC00017366/detail
		Shubuta, MS US	USC00228053	Daily	ncdc.noaa.gov/cdo-web/datasets/GHCND/stations/GHCND:USC00228053/detail
		Talladega, AL US	USC00018024	Daily	ncdc.noaa.gov/cdo-web/datasets/GHCND/stations/GHCND:USC00018024/detail
		Woodbury, GA US	USC00099506	Daily	ncdc.noaa.gov/cdo-web/datasets/GHCND/stations/GHCND:USC00099506/detail
	NOAA	Global Precipitation Climatology Center			esrl.noaa.gov/psd/data/gridded/data.gpcc.html
		LTM Total Full V2018	--	0.5 degree*	ftp.cdc.noaa.gov/Datasets/gpcc/full_v2018/precip.mon.ltm.v2018.nc
Discharge	USGS	National Water Information System			waterdata.usgs.gov
		Alabama River at Claiborne, AL	02429500	Daily	waterdata.usgs.gov/nwis/inventory/?site_no=02429500&agency_cd=USGS
		Alabama River at Claiborne…	02428400	Daily	waterdata.usgs.gov/nwis/inventory/?site_no=02428400&agency_cd=USGS
		Apalachicola River at Chatt…	02358000	Daily	waterdata.usgs.gov/nwis/inventory/?site_no=02358000&agency_cd=USGS
		Pascagoula River at Merrill, MS	02479000	Daily	waterdata.usgs.gov/nwis/inventory/?site_no=02479000&agency_cd=USGS
		Tombigbee R at Coffeeville…	02469761	Daily	waterdata.usgs.gov/nwis/inventory/?site_no=02469761&agency_cd=USGS
		Tombigbee River near Leroy, AL	02470000	Daily	waterdata.usgs.gov/nwis/inventory/?site_no=02470000&agency_cd=USGS
Water level	NOAA	Tides & Currents			tidesandcurrents.noaa.gov
		Dauphin Island, AL	8735180	Hourly	tidesandcurrents.noaa.gov/stationhome.html?id=8735180#info
	USGS	National Water Information System			waterdata.usgs.gov
		Mobile River at river mile 31.0…	02470629	30 min	waterdata.usgs.gov/nwis/inventory/?site_no=02470629&agency_cd=USGS
		Tombigbee R bl Coffeeville…	02469762	Hourly	waterdata.usgs.gov/nwis/inventory?agency_code=USGS&site_no=02469762
		Tombigbee River at steamplant…	02470050	Hourly	waterdata.usgs.gov/nwis/inventory/?site_no=02470050&agency_cd=USGS
Climate Indicies	NOAA	Global Climate Observing System			esrl.noaa.gov/psd/gcos_wgsp/Timeseries
		Atlantic multidecadal oscillation	AMO	Monthly	esrl.noaa.gov/psd/data/correlation//amon.us.long.data
		AMO "not detrended"	AMO (trend)	Monthly	esrl.noaa.gov/psd/data/correlation/amon.us.long.mean.data
		Dipole Mode Index	DMI	Monthly	esrl.noaa.gov/psd/gcos_wgsp/Timeseries/Data/dmi.long.data
		Niño 1+2 SST Index	Niño 1+2	Monthly	esrl.noaa.gov/psd/gcos_wgsp/Timeseries/Data/nino12.long.data
		Niño 3 SST Index	Niño 3	Monthly	esrl.noaa.gov/psd/gcos_wgsp/Timeseries/Data/nino3.long.data
		Niño 3.4 SST Index	Niño 3.4	Monthly	esrl.noaa.gov/psd/gcos_wgsp/Timeseries/Data/nino34.long.data
		Niño 4 SST Index	Niño 4	Monthly	esrl.noaa.gov/psd/gcos_wgsp/Timeseries/Data/nino4.long.data
		North Atlantic Oscillation	NAO	Monthly	esrl.noaa.gov/psd/gcos_wgsp/Timeseries/Data/nao.long.data
		North Pacific Index	NPI	Monthly	esrl.noaa.gov/psd/gcos_wgsp/Timeseries/Data/np.long.data
		Pacific Decadal Oscillation	PDO	Monthly	esrl.noaa.gov/psd/gcos_wgsp/Timeseries/Data/pdo.long.data
		Pacific North American Index	PNA	Monthly	esrl.noaa.gov/psd/data/20thC_Rean/timeseries/monthly/PNA/pna.20crv2c.long.data
		Southern Annular Mode	SAM	Monthly	esrl.noaa.gov/psd/data/20thC_Rean/timeseries/monthly/SAM/sam.20crv2c.long.data
		Southern Oscillation Index	SOI	Monthly	esrl.noaa.gov/psd/gcos_wgsp/Timeseries/Data/soi.long.data
		Trans Polar Index	TPI	Monthly	esrl.noaa.gov/psd/gcos_wgsp/Timeseries/Data/tpi.long.data
Watershed	USGS	Watershed Boundary Dataset			
		2-digit Hydrologic Unit - 03	--	--	sciencebase.gov/catalog/item/5a1632b3e4b09fc93dd171e2
Land Cover	USGS	Maryland Water Science Center			
		Wieczorek et al. 2018	--	10 years	sciencebase.gov/catalog/item/58cbeef2e4b0849ce97dcd61
Rating Curves	USGS	Rating Curve Builder	various+		waterwatch.usgs.gov/?id=mkrc
	Peer-Reviewed Literature				
		Dykstra & Dzwonkowski 2020	--		data.gulfresearchinitiative.org/data/R4.x260.000:0125

*Resolution is spatial

+Station IDs used for discharge rating curves: 02479310 (Graham Ferry), 02479000 (Merrill), 02359170 (Sumatra), 02358700 (Blountstown

Table S2. Discharge and precipitation (a) p-values and (b) correlations with climate indices. Results were calculated from monthly averages using Kendall's tau. For full names of indices, see Table S1.

a

Climate Oscillation	Discharge Pascagoula	Discharge Tom-AL	Discharge Apalachicola	Precipitation Pascagoula	Precipitation Tom-AL	Precipitation Apalachicola
AMO	0.00	0.00	0.00	0.04	0.10	0.07
AMO (trend)	0.00	0.00	0.00	0.20	0.15	0.34
DMI	0.86	0.12	0.94	0.42	0.39	0.35
Niño 1+2	0.00	0.00	0.00	0.39	0.05	0.60
Niño 3	0.00	0.00	0.00	0.82	0.07	0.09
Niño 3.4	0.00	0.00	0.00	0.71	0.18	0.06
Niño 4	0.24	0.06	1.00	0.65	1.00	0.24
NAO	0.00	0.00	0.01	0.75	0.68	0.50
NPI	0.00	0.00	0.02	0.00	0.04	0.00
PDO	0.00	0.23	0.06	0.63	0.91	0.62
PNA	0.63	0.53	0.67	0.94	0.82	0.78
SAM	0.04	0.10	0.00	0.24	0.96	0.62
SOI	0.06	0.18	0.02	0.20	0.37	0.34
TPI	0.01	0.02	0.00	0.71	0.84	0.35

b

Climate Oscillation	Discharge Pascagoula	Discharge Tom-AL	Discharge Apalachicola	Precipitation Pascagoula	Precipitation Tom-AL	Precipitation Apalachicola
AMO	-0.06	-0.09	-0.10	-0.04	-0.04	-0.04
AMO (trend)	-0.39	-0.44	-0.35	0.03	-0.03	0.02
DMI	0.00	0.03	0.00	0.02	0.02	0.02
Niño 1+2	0.41	0.44	0.38	-0.02	0.04	0.01
Niño 3	0.32	0.34	0.33	-0.01	0.04	0.04
Niño 3.4	0.15	0.16	0.19	0.01	0.03	0.04
Niño 4	-0.02	-0.04	0.00	0.01	0.00	0.03
NAO	0.07	0.08	0.06	-0.01	-0.01	0.01
NPI	-0.08	-0.11	-0.05	0.08	0.04	0.09
PDO	0.07	0.02	0.04	-0.01	0.00	0.01
PNA	-0.01	-0.01	0.01	0.00	-0.01	-0.01
SAM	-0.04	-0.03	-0.08	0.03	0.00	-0.01
SOI	-0.04	-0.03	-0.05	-0.03	-0.02	-0.02
TPI	0.05	0.05	0.09	-0.01	0.00	0.02

Table S3. (a) P-values and (b) correlation between climate indices. Results were calculated from monthly averages using Kendall's tau. For full names of indices, see Table S1.

a

Osillation	AMO	AMO (tre	DMI	Niño 1+2	Niño 3	Niño 3.4	Niño 4	NAO	NPI	PDO	PNA	SAM	SOI	TPI
AMO	0.00	0.00	0.14	0.01	0.55	0.23	0.00	0.00	0.05	0.23	0.30	0.35	0.93	0.31
AMO (trend)	0.00	0.00	0.91	0.00	0.00	0.00	0.00	0.00	0.00	0.11	0.57	0.00	0.43	0.31
DMI	0.14	0.91	0.00	0.00	0.00	0.00	0.00	0.15	0.33	0.01	0.45	0.00	0.00	0.00
Niño 1+2	0.01	0.00	0.00	0.00	0.00	0.00	0.14	0.02	0.00	0.00	0.12	0.01	0.00	0.00
Niño 3	0.55	0.00	0.00	0.00	0.00	0.00	0.00	0.69	0.87	0.00	0.00	0.00	0.00	0.00
Niño 3.4	0.23	0.00	0.00	0.00	0.00	0.00	0.00	0.05	0.00	0.00	0.00	0.00	0.00	0.00
Niño 4	0.00	0.00	0.00	0.14	0.00	0.00	0.00	0.02	0.00	0.00	0.00	0.04	0.00	0.00
NAO	0.00	0.00	0.15	0.02	0.69	0.05	0.02	0.00	0.65	0.54	0.00	0.54	0.34	0.12
NPI	0.05	0.00	0.33	0.00	0.87	0.00	0.00	0.65	0.00	0.00	0.00	0.09	0.00	0.00
PDO	0.23	0.11	0.01	0.00	0.00	0.00	0.00	0.54	0.00	0.00	0.00	0.88	0.00	0.00
PNA	0.30	0.57	0.45	0.12	0.00	0.00	0.00	0.00	0.00	0.00	0.00	0.86	0.00	0.00
SAM	0.35	0.00	0.00	0.01	0.00	0.00	0.04	0.54	0.09	0.88	0.86	0.00	0.00	0.00
SOI	0.93	0.43	0.00	0.00	0.00	0.00	0.00	0.34	0.00	0.00	0.00	0.00	0.00	0.00
TPI	0.31	0.31	0.00	0.00	0.00	0.00	0.00	0.12	0.00	0.00	0.00	0.00	0.00	0.00

b

Osillation	AMO	AMO (tre	DMI	Niño 1+2	Niño 3	Niño 3.4	Niño 4	NAO	NPI	PDO	PNA	SAM	SOI	TPI
AMO	1.00	0.21	0.03	-0.05	-0.01	0.02	0.07	-0.08	0.04	0.02	0.02	0.02	0.00	-0.02
AMO (trend)	0.21	1.00	0.00	-0.66	-0.34	-0.07	0.15	-0.07	0.32	-0.03	0.01	0.06	-0.02	-0.02
DMI	0.03	0.00	1.00	0.08	0.11	0.12	0.13	-0.03	-0.02	-0.05	-0.02	0.07	-0.12	0.08
Niño 1+2	-0.05	-0.66	0.08	1.00	0.61	0.30	0.03	0.05	-0.20	0.14	0.03	-0.06	-0.11	0.20
Niño 3	-0.01	-0.34	0.11	0.61	1.00	0.66	0.32	-0.01	0.00	0.24	0.10	-0.06	-0.26	0.40
Niño 3.4	0.02	-0.07	0.12	0.30	0.66	1.00	0.61	-0.04	0.09	0.27	0.16	-0.08	-0.39	0.57
Niño 4	0.07	0.15	0.13	0.03	0.32	0.61	1.00	-0.05	0.06	0.27	0.16	-0.04	-0.43	0.58
NAO	-0.08	-0.07	-0.03	0.05	-0.01	-0.04	-0.05	1.00	-0.01	-0.01	-0.06	-0.01	0.02	-0.03
NPI	0.04	0.32	-0.02	-0.20	0.00	0.09	0.06	-0.01	1.00	-0.11	-0.28	0.04	0.06	-0.09
PDO	0.02	-0.03	-0.05	0.14	0.24	0.27	0.27	-0.01	-0.11	1.00	0.27	0.00	-0.22	0.43
PNA	0.02	0.01	-0.02	0.03	0.10	0.16	0.16	-0.06	-0.28	0.27	1.00	0.00	-0.14	0.22
SAM	0.02	0.06	0.07	-0.06	-0.06	-0.08	-0.04	-0.01	0.04	0.00	0.00	1.00	0.07	-0.08
SOI	0.00	-0.02	-0.12	-0.11	-0.26	-0.39	-0.43	0.02	0.06	-0.22	-0.14	0.07	1.00	-0.46
TPI	-0.02	-0.02	0.08	0.20	0.40	0.57	0.58	-0.03	-0.09	0.43	0.22	-0.08	-0.46	1.00

References

U.S. Bureau of Public Roads (1952), Highway Statistics 1950, U.S. 6th annual series, U.S. Department of Commerce, Washington 25, DC.

U.S. Bureau of Public Roads (1962), Highway Statistics 1960, 16th annual series, U.S. Department of Commerce, Washington 25, DC.

U.S. Federal Highway Administration (1972), Highway Statistics 1980, 26th annual series, U.S. Department of Transportation, Washington, DC.

U.S. Federal Highway Administration (1982), Highway Statistics 1980, 36th annual series, U.S. Department of Transportation, Washington, DC.

U.S. Federal Highway Administration (1992), Highway Statistics 1990, 46th annual series, U.S. Department of Transportation, Washington, DC.

U.S. Federal Highway Administration (2003), Highway Statistics 2000, 56th annual series, U.S. Department of Transportation, Washington, DC.

U.S. Federal Highway Administration (2014), Highway Statistics 2010, 66th annual series, U.S. Department of Transportation, Washington, DC.

U.S. Federal Highway Administration (2021), Highway Statistics 2018, 74th annual series, U.S. Department of Transportation, Washington, DC.

U.S. Census Bureau (2021). *Historical Population Density Data (1910-2020).* Retrieved from [https://www.census.gov/data/tables/time-series/dec/density-data-text.html].
